# High-resolution lithographic biofabrication of hydrogels with complex microchannels from low-temperature-soluble gelatin bioresins

**DOI:** 10.1016/j.mtbio.2021.100162

**Published:** 2021-11-19

**Authors:** Riccardo Levato, Khoon S. Lim, Wanlu Li, Ane Urigoitia Asua, Laura Blanco Peña, Mian Wang, Marc Falandt, Paulina Nuñez Bernal, Debby Gawlitta, Yu Shrike Zhang, Tim B.F. Woodfield, Jos Malda

**Affiliations:** a Department of Clinical Sciences, Faculty of Veterinary Medicine, Utrecht University, the Netherlands; b Department of Orthopaedics, University Medical Center Utrecht, the Netherlands; c Christchurch Regenerative Medicine and Tissue Engineering (CReaTE) Group, Department of Orthopaedic Surgery and Musculoskeletal Medicine, University of Otago Christchurch, Christchurch, the Netherlands; d Division of Engineering in Medicine, Department of Medicine, Brigham and Women's Hospital, Harvard Medical School, Cambridge, USA; e Department of Oral and Maxillofacial Surgery & Special Dental Care, University Medical Center Utrecht, the Netherlands

**Keywords:** Biofabrication and bioprinting, Lithography, Bioresin, Hydrogel, Digital light processing

## Abstract

Biofabrication via light-based 3D printing offers superior resolution and ability to generate free-form architectures, compared to conventional extrusion technologies. While extensive efforts in the design of new hydrogel bioinks lead to major advances in extrusion methods, the accessibility of lithographic bioprinting is still hampered by a limited choice of cell-friendly resins. Herein, we report the development of a novel set of photoresponsive bioresins derived from ichthyic-origin gelatin, designed to print high-resolution hydrogel constructs with embedded convoluted networks of vessel-mimetic channels. Unlike mammalian gelatins, these materials display thermal stability as pre-hydrogel solutions at room temperature, ideal for bioprinting on any easily-accessible lithographic printer. Norbornene- and methacryloyl-modification of the gelatin backbone, combined with a ruthenium-based visible light photoinitiator and new coccine as a cytocompatible photoabsorber, allowed to print structures resolving single-pixel features (∼50 ​μm) with high shape fidelity, even when using low stiffness gels, ideal for cell encapsulation (1–2 ​kPa). Moreover, aqueous two-phase emulsion bioresins allowed to modulate the permeability of the printed hydrogel bulk. Bioprinted mesenchymal stromal cells displayed high functionality over a month of culture, and underwent multi-lineage differentiation while colonizing the bioresin bulk with tissue-specific neo-deposited extracellular matrix. Importantly, printed hydrogels embedding complex channels with perfusable lumen (diameter <200 ​μm) were obtained, replicating anatomical 3D networks with out-of-plane branches (*i.e.* brain vessels) that cannot otherwise be reproduced by extrusion bioprinting. This versatile bioresin platform opens new avenues for the widespread adoption of lithographic biofabrication, and for bioprinting complex channel-laden constructs with envisioned applications in regenerative medicine and hydrogel-based organ-on-a-chip devices.

## Graphical abstract

**Figure:**
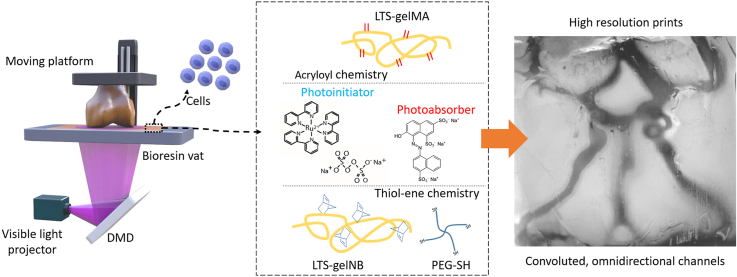

## 1 Introduction

The automation of the *in vitro* production of living tissues and the generation of clinically relevant, centimeter-scale constructs remains a major hope towards the availability of implantable grafts for regenerative medicine, as well as for manufacturing advanced *in vitro* models for drug discovery [1].

The intricate structure of biological tissue is a result of a precise sequence of cell-driven processes occurring during tissue development, which, in a laboratory settings, to date can only be partially recapitulated within miniaturized-scale structures originated by stem cells, such as organoids [2]. Conversely, reconstructing functional biological constructs through an engineering- and design-driven approach, in which biomaterials and cell-based building blocks can be assembled in a spatially controlled fashion, remains a major challenge. In native tissues and organs, biological function is intimately linked to tissue structure and architecture, in which different extracellular matrix components and cells are organized [3]. The recent advances in biofabrication technologies, which comprise the rapidly evolving field of three-dimensional (3D) bioprinting [4], are aiding material scientists, biologists and engineers to recapitulate more closely the spatial patterning found in native tissues, and thus producing, in a laboratory settings, a new generation of cell-laden, three-dimensional constructs that can mimic salient functions of native organs [3,5].

In the past decade, most engineering and (bio)material development efforts in the field of biofabrication had been centered on extrusion-based bioprinting techniques. These involve dispensing a cell-laden suspension, termed bioink, through a nozzle which is used to build the desired object in 3D in a layer-by-layer fashion. Among the different classes of materials applied in 3D printing, bioinks are most often based on hydrogel precursors, as these provide a water-rich environment suitable to preserve cell viability during the printing process, as well as to sustain cell function post-printing, offering a 3D environment for tissue culture [6,7]. Although highly versatile, especially when aiming to print multiple materials [8], extrusion techniques generally offer a minimum resolution (≈100 ​μm) limited by a compromise between the nozzle diameter and the maximum shear stress that the cells can tolerate [9]. Moreover, since in most extrusion-based printers objects are built by piling dispensed hydrogel fibers on top each other layer-by-layer, resolving convoluted geometries, like woven patterns or intricate tubular networks, such as those found in several segments of the mammalian vascular system, remains a major challenge [10]. Reconstructing vascular analogues is of particular importance to ensure nutrient supply to large, centimeter-scale constructs. In this perspective, advanced biofabrication technologies hold the potential to recreate channels that mimic afferent and efferent vessels in terms of size and architecture, at a length scale that the spontaneous self-assembly of endothelial cells into micron-sized capillaries cannot otherwise reach [11].

Only recently, increasing attention has been directed towards the development of nozzle-free, light-based bioprinting techniques that hold an untapped potential to overcome the abovementioned limitations. Derived from conventional additive manufacturing techniques based on vat polymerization such as stereolithography [12], light-based bioprinting, involves the use of cell-laden, photocrosslinkable hydrogel precursor solutions, also termed bioresins, that are exposed to spatially coordinated light patterns. These patterns are most often produced by laser scanning or *via* digital micromirror devices (DMDs), triggering a locally controlled polymerization of the bioresin into the desired 3D object one layer at a time. By controlling the voxel size during the photo-exposure steps, high resolution, superior to those of common extrusion techniques can be achieved, typically in the range of 1–50 ​μm [13,14], and with a freedom of design that can easily resolve even convoluted microvascular-like networks [15]. Current limitations in light-based printing include the loss of cells together with the unreacted bioresin volume, and the inherent difficulties in printing with multiple resins in the same process, although elegant strategies combining microfluidics and digital light projection (DLP) bioprinting have also been introduced to allow for multi-material fabrication [16]. Moreover, light-based bioprinting uniquely enables the fabrication of features at even submicron-resolution, when adopting multiphoton polymerization [17]. Furthermore, recent developments have also demonstrated ultra-fast volumetric bioprinting of complex centimeter-scale 3D cell-laden constructs in less than 30 ​s, by tomographic light patterning [18]. Despite such promising advances, only few hydrogel-based bioresins are currently available, the vast majority of which are based on synthetic polymers, prevalently acrylate-derivatives of polyethylene glycol, which, in their native form, lack cell instructive cues [15,[19], [20], [21], [22]]. On the other hand, natural-origin polymers have limited capacity to enable bioprinting with high resolution and shape fidelity. Such hydrogel-forming materials are prevalently used at low polymer concentration in order to form structures with low stiffness suitable for optimal cell culture, but thus with limited ability to preserve the geometry imposed by the printing process. Among natural hydrogels, photo-responsive gelatin derivatives are widely studied across a range of biofabrication techniques, due to their favorable biological performance in terms of cell adhesion domains and biodegradability. Most works focused on extrusion printing techniques, in which printability and shape fidelity are primarily governed by the rheological properties of the bioinks, particularly their viscosity, the presence of a yield stress, a marked shear-thinning profile and quick shear recovery kinetics, and quantitative descriptions of these parameters have been extensively covered in the literature [[23], [24], [25]]. In such applications, the well-described thermo-sensitive behavior of gelatin is advantageous. On the contrary, bioresins for light-based printing display different rheological requirements. In fact, printability is mainly dependent on photochemical properties, which determine reactivity, resolution and shape fidelity of the printed construct [26], and low-viscosity materials (typically 0.25–10 ​Pa ​s [27]) are preferred instead of polymers that gelate around room temperature. Thus, developing a new range of bioresins that preserve the inherent bioactivity of gelatin, while permitting ease of application for lithography-based bioprinting and maximal resolution, could open new possibilities for the field of biofabrication.

This work describes a novel strategy to bioprint high-resolution living constructs featuring embedded free-form microfluidic, vessel-like channels, permitted by the development of a thermostable hydrogel-based platform for DLP bioprinting, that can be used on any DLP device. Photo-responsive ichthyic-origin gelatins, displaying low melting point and bearing acryloyl and norbornene groups, and which span a wide array of mechanical properties were developed as visible-light responsive bioresins for DLP printing. A particular attention was directed towards designing a system to enable prints with high shape fidelity microchannels, even when using hydrogels displaying very low stiffness (∼1 ​kPa). Such system would overcome the current limitations experience in the field of DLP (bio)printing, which is mostly reliant on stiff high polymer content materials [28], and thus can open new opportunities for material scientists, engineers and biologists to build the next generation of 3D constructs with ideal properties for cell encapsulation, tissue engineering and regenerative medicine. The DLP working curve for the low-temperature soluble gelatin-based bioresins, exposure settings and printing resolution were described as a function of crosslinking chemistry and the polymer concentration. To maximize printing resolution, an array of biocompatible food dyes were studied as photoabsorbers to modulate the spatio-selective crosslinking of the gelatin derivatives. The resulting bioresins were studied for their potential to sustain long-term functionality and differentiation capacity of bioprinted stem cells. Furthermore, the bioresins allowed the production of in-hydrogel perfusable microvascular-mimetic networks branching in the x, y and z directions. Finally, together with this superior printing resolution and freedom of design, further modification of the bioresin composition was also investigated in order to produce hydrogel-built structures with tunable bulk diffusion properties.

## 2 Experimental section

### 2.1 Macromere synthesis and hydrogel preparation

Low temperature soluble (LTS) bioresins were synthesized starting from gelatin from cold water fish skin (Sigma-Aldrich, the Netherlands). For the synthesis of gelatin methacryloyl (GelMA), an adapted version of a previously published protocol was followed [29]. Briefly, the LTS gelatin was dissolved in phosphate-buffered saline (PBS) to form a 10% w/v solution, and it was reacted with 1:0.6 methacrylic anhydride (Sigma-Aldrich, The Netherlands) at 37 ​°C for 1 ​h, to obtain an ≈90% degree of modification of the lysine residues (Supplementary Figs. S1 and S2). The quantification of the degree of modification was performed according to a previous published protocol [30], and further details are reported in the Supplementary Information. Gelatin norbornene (GelNB), able to undergo thiol-ene photo-click crosslinking, and thus enabling the fabrication of more controlled hydrogel networks when compared to chain polymerization [31], was also obtained through a synthesis protocol based on previous reports [32,33]. Gelatin (10% w/v) was dissolved in PBS and supplemented with a 20% w/v carbic anhydride (Acros Organics, Belgium). The pH of the mixture was continuously adjusted to 7.5–8.0 using 1 ​M NaOH, as the carbic anhydride dissolved. Once the pH stabilized, the reaction was left to run in the dark for 24 ​h, to obtain a final degree of substitution of ≈85% (Supplementary Figs. S1 and S3), after which it was quenched by a 1:3 dilution with PBS. For both LTS-GelMA and -GelNB, excess of the respective anhydride was removed by centrifugation, and the remaining solutions were neutralized, dialyzed against distilled water (MW_cut-off_ ​= ​12,000 ​Da), sterile-filtered, freeze-dried and stored at −20 ​°C until used. To prepare the different hydrogels, LTS-GelMA and -GelNB were dissolved in PBS at the desired concentration, in presence of 2 ​mM tris(2,2′-bipyridyl)ruthenium (II) chloride hexahydrate (Acros Organics, Belgium) and 20 ​mM sodium persulfate (Acros Organics, Belgium) (Ru/SPS), which acted as visible light photoinitiator able to minimize the effect of oxygen inhibition on photocrosslinking, as previously demonstrated [14,34]. In the case of step growth polymerizable LTS-GelNB, a tetra-thiol polyethylene glycol-based crosslinker (4-arm PEG-SH, Mw ​= ​5000 ​Da, JenKem Technology, USA) was added to achieve a 1:1 ​M ratio of thiol groups to the norbornene groups from the gelatin backbone. Macromer solutions were then exposed to a visible light lamp (600 lumen, λ ​= ​400–700 ​nm, I ​= ​6.5 ​mW/cm^2^ at 450 ​nm and at a distance of 10 ​cm) for 7.5 ​min to crosslink into the desired gelatin-based hydrogels.

### 2.2 Physico-chemical characterization of the hydrogels

Gelatin hydrogel precursor solutions at different polymer concentrations were casted into custom-made polydimethylsiloxane (PDMS) molds and photocrosslinked to obtain cylindrical samples (diameter ​= ​5 ​mm, height ​= ​1 ​mm). Samples (n ​= ​3) were weighed to obtain the initial wet weight (*m*_in *t* ​= ​0_) or lyophilized overnight first, to obtain their dry weights (*m*_dry__*t* ​= ​0_). These samples were then incubated in PBS at 37 ​°C overnight, freeze-dried and weighed (m_dry_). The sol fraction was then calculated *via* Equation (1):

(1) $$\%\;sol\;fraction=\frac{m_{dry\;t=0}-\;m_{dry}}{m_{dry\;t=0}}\times 100$$

The mechanical properties of the hydrogels were measured in an unconfined, uniaxial compression test, using a dynamic mechanical analyzer (DMA, Q800, TA Instruments, USA). Cylindrical samples (n ​= ​3) were incubated in PBS overnight, and subsequently subjected to a strain ramp (−20% strain/minute, preload 1% strain). The compression modulus was calculated from the slope of the stress-strain curve in the 10–15% strain region.

### 2.3 Bioresin formulations, effect of photoabsorbers and DLP working curve

The UV–vis absorption spectra of the Ru/SPS mixture and several photoabsorbers in the 300–650 ​nm region were obtained using a spectrophotometer. Candidate photoabsorbers were new coccine (red, Queen Fine Foods, Australia), a mixture of tartrazine and azorubine (yellow, Queen Fine Foods, Australia), and fast green (Sigma-Aldrich, the Netherlands). Dyes that attenuated but not entirely blocked light transmission in the spectral region coinciding with the absorption peak of Ru/SPS and with the main emission peak of the light source used in the DLP printer (Perfactory 3 Mini, Envisiontec, Germany) were selected for bioprinting. Next, bioresins composed of Ru/SPS, the selected photoabsorber at varying concentrations, and either LTS-GelMA or -GelNB, were loaded onto a glass slide placed on the vat of the printer, and irradiated to print disc shaped samples (diameter ​= ​4 ​mm), exposed to increasing irradiation doses (n ​= ​3 per dose). The curing depth (C_d_) was obtained measuring the height of each disk using a stereomicroscope. The height of the disks, equivalent to C_d_ was plotted as a function of the respective irradiation energy. The DLP working curve was thus obtained as the linear relation between a given dose (expressed in logarithmic scale) and C_d_. The curve is also defined by equation (2), with D_p_ being the light penetration depth, and E_c_ the critical energy to reach the gel point:

(2) $$C_{d}=D_{p}\cdot ln\frac{E}{E_{c}}$$

For each bioresin formulation, D_p_ can thus be obtained as the slope of the working curve, and E_c_ is therefore obtained by solving equation (2), as previously described [14].

### 2.4 DLP bioprinting

#### 2.4.1 Cell harvesting, printing and viability

**Fig. 1:**
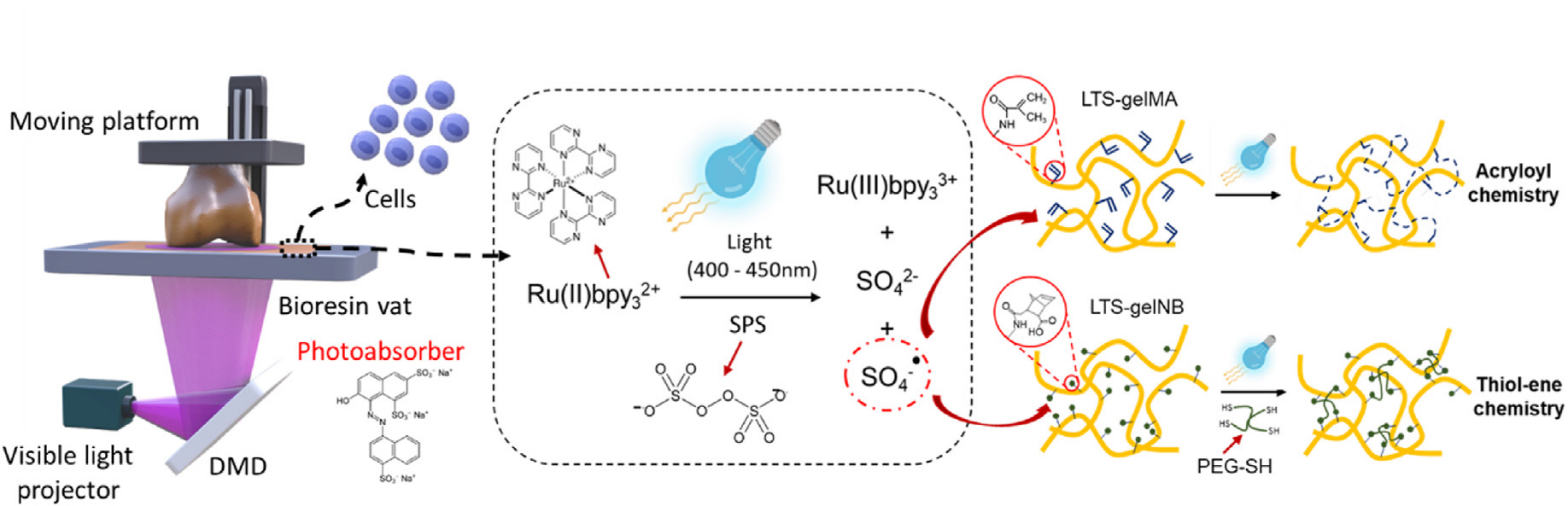
Schematic overview of the DLP-based bioprinting process and of the composition of the two LTS gelatin-based bioresins, including a representation of the photoinitiator response to light and the resulting crosslinking reaction.

Bone marrow-derived mesenchymal stromal cells (MSCs) were obtained post-mortem from the sternum of a skeletally mature equine donor (age ​= ​5 years old), as previously described [35]. The horse was donated to science by its owner, in accordance to the ethical guidelines of the Faculty of Veterinary Medicine of Utrecht University. Isolated MSCs were cultured in expansion media, consisting of in alpha-MEM (Life Technologies, the Netherlands), supplemented with 10% FCS, 100 U/mL of penicillin, 100 ​*μ*g/mL of streptomycin, 0.2 ​mM of L-ascorbic acid-2-phosphate and 1 ​ng/mL of basic fibroblast growth factor (bFGF, R&D Systems, USA). Cells were used at passage 4 to 6 for all experiments. A schematic representation of the bioresin composition is depicted in Fig. 1. For DLP bioprinting, MSCs were suspended in the bioresin formulations (supplemented at a concentration of 10^7^ ​cells/mL). Constructs were printed with optimized settings based on the data derived from the working curve, using a layer height of 50 ​*μ*m, with a power density of 6.5 ​mW/cm^2^ at the surface of the vat, and 10 ​s of exposure time per each layer. After printing, cell-laden constructs (n ​= ​3 per condition and time point) were kept in culture in expansion media, and viability was assessed at day 1 and 7, using a live/dead assay, using 1 ​*μ*g/mL of calcein AM and ethidium homodimer-1 (Life Technologies, the Netherlands). For each construct, 5 random sections were imaged using a fluorescence microscope (BX51 Olympus, the Netherlands), and the amount of living cells were manually counted using ImageJ.

#### 2.4.2 Printing resolution and bioresin stability over time

To characterize the printing resolution, a calibration structure consisting of an array of positive (comb-like posts) and negative (hollow channels and spacing) features of decreasing size was printed. Optical micrographs of these prints (n ​= ​3 per condition), obtained from both LTS-GelMA 10% w/v and LTS-GelNB 5% w/v were captured, and the characteristic dimensions of the positive and negative features measured with ImageJ and compared to the original computer design. Next, the stability of the bioresin upon mixing with the photoinitiators was assessed by printing disc shaped constructs (diameter ​= ​5 ​mm, height ​= ​1 ​mm, n ​= ​3) after an increasing latency time. Upon addition of Ru/SPS, LTS-GelMA and LTS-GelNB were kept in the dark and photoexposed after 0, 15, 30, 45 or 60 ​min. The efficiency of crosslinking was estimated measuring the compressive modulus of the discs, following the procedure described in Section 2.2.

#### 2.4.3 Long-term cell function post-printing

To assess the ability of the printed bioresin to provide a permissive environment for cell differentiation, MSC-laden (10^7^ ​cells/mL) LTS-GelMA disc-shaped constructs (n ​= ​3 per culture condition) were bioprinted, and cultured for 28 days in either *i*) osteogenic medium (α-Minimum Essential Medium (MEM), supplemented with 10% FCS, 100 U/mL of penicillin, 100 ​*μ*g/mL of streptomycin, 0.2 ​mM of L-ascorbic acid-2-phosphate, 20 ​mM of *β*-glycerophosphate, 10 ​nM of dexamethasone); or *ii*) chondrogenic medium (Dulbecco's Modified Eagle Medium (DMEM), supplemented with 1% insulin-transferrin-selenous acid premix (ITS-premix, Corning), 100 U/mL of penicillin, 100 ​*μ*g/mL of streptomycin, 0.2 ​mM of L-ascorbic acid-2-phosphate, 100 ​nM of dexamethasone, 10 ​ng/mL of human recombinant transforming growth factor *β*1 (TGF-*β*1, PeproTech, UK)); or *iii*) under a hypertrophic differentiation regime, consisting of 14 days of exposure to chondrogenic media, followed by 14 days of exposure to hypertrophy induction media (DMEM, 1% ITS ​+ ​premix, 10^−9^ ​M of dexamethasone, 10 ​mM of *β*-glycerophosphate, 0.2 ​mM of L-ascorbic acid 2-phosphate, 100 U/mL of penicillin, 100 ​*μ*g/mL of streptomycin, 1 ​nM of 3,3′,5-triiodo-L-thyronine (T3, Sigma-Aldrich, the Netherlands)) [36]. After 28 days, samples were cut into 3 parts for: histological processing, quantification of sulphated glycosaminoglycans (sGAGs) and quantification of alkaline phosphatase activity (ALP). For histological processing, the samples were fixed in neutral buffered formalin, dehydrated in a graded ethanol series, cleared with xylene and embedded in paraffin. Microtome slices (10 ​μm thick) were obtained, and used for immunohistochemical staining. Immunostaining for collagen type II, performed according to a previously published protocol [35], a hallmark ECM component from cartilage was used as a qualitative marker for chondrogenic differentiation, while the von Kossa staining [36] was used to detect the presence of mineralized matrix, characteristic of MSC commitment towards osteogenesis or hypertrophic chondrogenesis. For the quantitative assessment of sGAGs, harvested samples were frozen and stored at −20 ​°C. Next, samples were thawed and digested with papain and sGAGs measured with a dimethylmethylene blue (DMMB) assay following previously published protocols [37]. Samples for ALP activity quantification were instead freshly ground with a pestle and incubated in mammalian protein extraction reagent (MPER, Life Technologies, the Netherlands). The obtained cell lysates were used to perform a p-nitrophenyl phosphate assay (SIGMAFAST, Sigma-Aldrich, the Netherlands), as previously reported [38]. Both sGAGs and ALP quantitative data were normalized against the DNA content of the hydrogels, as determined using the Quant-iT-Picogreen-dsDNA kit (Molecular Probes, Invitrogen, USA).

#### 2.4.4 Bioprinting of complex and perfusable vascular-like networks

A series of LTS-GelMA hydrogel-made models, embedding vascular-like channels of increasing geometrical complexity were printed and manually perfused to assess the highest resolution achievable when producing such structures, and to demonstrate the ability to generate branched channels extending into the x-y-z directions. Structures were designed in Tinkercad (Autodesk, USA) and converted to STL files to be used in the DLP printer. First, an array of straight channels with decreasing lumen diameter (1 ​mm–0.1 ​mm) and perpendicular to the direction of the light projected by the printer were built. Samples where perfused by manually pipetting a dyed aqueous solution (Queen Red food dye, Queen Fine Foods, Australia), and imaged with a stereomicroscope to identify the smallest resolvable channels. Next, several microfluidics-inspired channels were printed, including a spiraling vessel wrapping around a straight vessel. In this structure, both channels were perfused with the radiopaque contrast agent MICROFIL (Flow Tech Inc., USA), and imaged via micro-computed tomography (μCT, Quantum FX-Perkin Elmer, voltage ​= ​90 ​kV, current ​= ​200 ​μA, voxel size ​= ​20 ​μm^3^, and total scanning time ​= ​3 ​min). Finally, a portion of the human cerebral arterial circle (Willis’ circle), as obtained from 3D angiographic images, was scaled down and reproduced within a printed hydrogel block. Given the complex and ramified structure of this vessel network, one entry and one exit tube were drawn and connected manually in the CAD software, to ensure the formation of a single open circuit with no dead ends. Samples were perfused as described above and imaged with a stereomicroscope (Olympus SZ61, Japan).

#### 2.4.5 DLP bioprinting of emulsion bioresins with enhanced permeability

To demonstrate the further possibility to introduce microporosity within the bioprinted hydrogel bulk, the bioresin formulation was modified to form an aqueous two-phase emulsion (ATPE) bioresin [[39], [40], [41]]. For the ATPE bioresin preparation, LTS-GelMA solution and porogen solution were dissolved in PBS separately. The pore-forming bioresin was then achieved by mixing the two solutions via pipetting, to reach the final LTS-GelMA concentration at 15% w/v and 1.6% w/v polyethylene oxide (PEO, Mw ​= ​300 ​kDa, Sigma-Aldrich, USA) as a porogen. This specific GelMA concentration was chosen based on our preliminary tests, which suggested that a lower concentration did not support faithful DLP bioprinting of 3D constructs when combined with the high microporosity introduced by PEO-driven phase separation. The visible light photoinitiator system (Ru/SPS), and the new coccine photoabsorber were added to complete the bioresin formulation. Next, the pore-forming bioresin was printed with a DLP-based 3D bioprinter, which was custom-built using a visible-light projector (ViewSonic PA503W, USA). The permeability of the bioprinted constructs was assessed by, printing a cubic construct with an embedded central channel, and injecting the water-soluble red dye into the channel at the rate of 100 ​μL/h, using a syringe pump (New Era Pump Systems, USA). The diffusion profiles were evaluated over time for samples bioprinted with the porous LTS-GelMA and the standard LTS-GelMA without supplementation with the pore-forming PEO. An inverted fluorescent microscope (Nikon, Japan) and ImageJ were used to record and analyze the diffusion profiles.

### 2.5 Statistical analyses

Each experiment was performed in three replicates (n ​= ​3). Quantitative results are reported as means ​± ​standard deviations. Statistical analyses were performed with GraphPad Prism 6.0 (GraphPad Software, USA). Single comparisons were performed with a Student's t-test. Multiple comparisons between the experimental groups were performed using a one-way ANOVA, with a Bonferroni post-hoc test. Assumption of normality was checked with a Shapiro-Wilk test. When samples with not normal distribution were found, non-parametric tests were used (Mann-Whitney and Kruskal-Wallis tests). Values of p ​< ​0.05 were considered statistically significant.

## 3 Results and discussion

### 3.1 Targeting hydrogel physico-mechanical properties for bioprinting applications

**Fig. 2:**
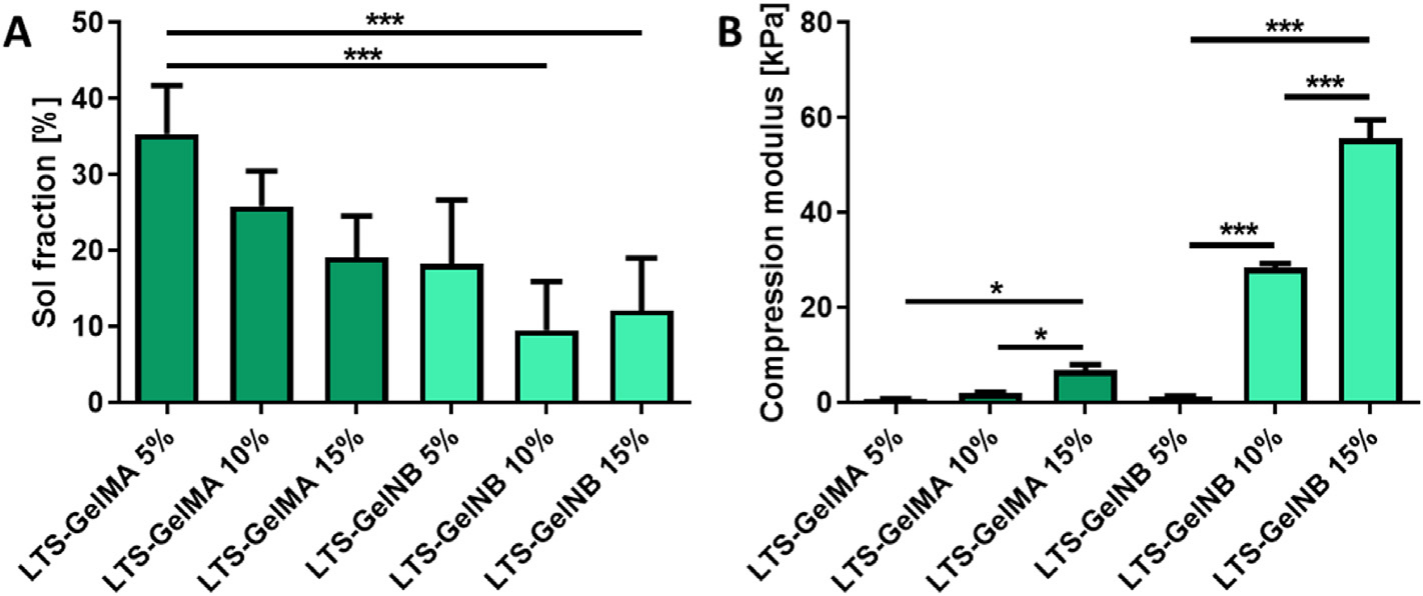
Physico-chemical characterizations of different hydrogel formulations. A) Mass loss analysis showing sol fraction values ranging between ∼10 and 30% and B) mechanical properties analyzed under uniaxial unconfined compression. While for both thiol-ene and acryloyl photochemistries the compressive modulus increased as expected via increasing the polymer content in the hydrogel precursor solutions, thiol-ene gelatins showed the ability to span a wider range of stiffness compared to acryloyl-based hydrogels. ∗ indicate statistically significant differences (∗ ​= ​p ​< ​0.05, ∗∗ ​= ​p ​< ​0.01, ∗∗∗ ​= ​p ​< ​0.001; n ​= ​3).

A family of gelatin-based hydrogels with varying polymer content were synthesized and characterized to produce bioresins able to display thermal stability in solution prior to crosslinking (Supplementary Fig. S4), to cover a broad range of mechanical properties suitable for cell encapsulation. First, all samples demonstrated a relatively low sol fraction (<40%, Fig. 2A), with no significant differences observed between LTS-GelMA and LTS-GelNB of various weight percentages. This result indicates that the crosslinking efficiency was similar for LTS-GelMA and LTS-GelNB hydrogels, despite them undergoing fundamentally different crosslinking mechanisms, namely chain-growth or step-growth reactions, respectively. However, only for the 5% w/v LTS-GelMA samples, the formed hydrogels demonstrated poor shape retention even upon gentle handling. Moreover, mechanical testing showed an obvious trend where increasing macromer concentration led to hydrogels of higher compressive moduli for both LTS-GelMA and LTS-GelNB (Supplementary Fig. S5). It was further observed that LTS-GelNB hydrogels showed higher tunability, where hydrogels over a wider range of compressive modulus (∼40-fold increase, 1.38 ​± ​0.05–55.63 ​± ​1.51 ​kPa, Fig. 2B) could be achieved by simply varying the macromer concentration from 5 to 15% w/v. In contrast, increasing LTS-GelMA from 5 to 15% w/v only resulted in an approximately 7-fold increase (0.99 ​± ​0.19–6.81 ​± ​1.20 ​kPa, Fig. 2B) in compressive stiffness. This evident difference between GelMA and GelNB even at comparable macromer concentration and sol fraction values can be explained by the fact that, in the LTS-GelNB samples, a non-zero length crosslinker needs to be incorporated in the forming hydrogel network. In this study, we selected as crosslinker 4-arm PEG-SH, with a Mw of 5000 ​Da, which is directly covalently bound in the hydrogel and thus likely contribute to the mechanical properties of the system, leading to the enhanced compressive modulus.

Interestingly, the moduli observed for LTS-GelMA appeared to be lower than previously reported values for porcine skin-derived GelMA, a difference likely due to the lower content of hydroxyproline present in fish-derived gelatin, which in turn results in triple helices with lower melting point and mechanical stability [42,43].

A paramount feature of cell-laden hydrogels for engineering connective tissues, in which extracellular matrix (ECM) components are predominant (such is the case for bone or cartilage), is their ability to permit colonization and integration of neo-synthesized ECM macromolecules throughout the whole volume of the hydrogel. Previous work extensively highlighted the impact of tuning hydrogel stiffness in order to achieve successful cell differentiation [44], and several published reports on cartilage tissue engineering demonstrated how hydrogels with a higher degree of crosslinking, resulted in reduced cartilage matrix deposition, mainly confined to the pericellular environment [45]. Although such a result is dependent on several physico-chemical aspects of the hydrogel network including the available free space, mesh size, diffusivity, mechanical properties and charge distribution, stiffness measurements generally offer a good indication for hydrogel performance. For example, previous work demonstrated an clear difference for GelMA-based gels with compressive stiffness below ∼50 ​kPa [46] resulting in a homogenous distribution of collagenous and glycosaminoglycan-rich matrix, and similar results in gels with a stiffness <5 ​kPa [47] for bone-like matrix distribution from encapsulated stem cells. As such, in this work, after excluding the 5% w/v GelMA group due to its lack of structural stability, we selected the bioresin formulations LTS-GelMA 10 and 15% and LTS-GelNB at 5 and 10% w/v for further characterizations, which yielded hydrogels with a stiffness within the 1–30 ​kPa range.

### 3.2 Constructing the working curve for LTS-GelMA and LTS-GelNB bioresin to enable high-resolution printing

**Fig. 3:**
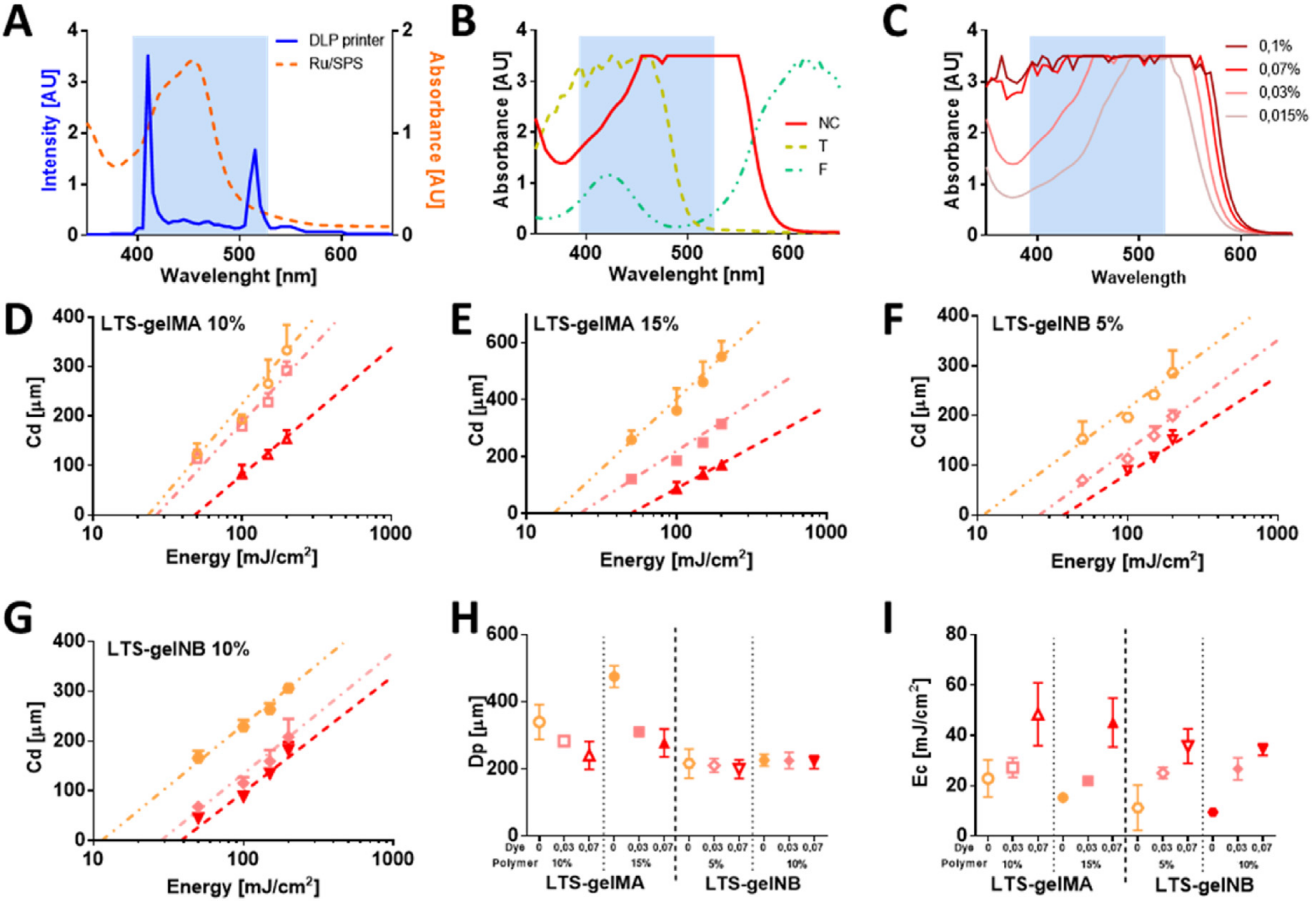
Identification of the working conditions for the DLP process suitable to ensure high resolution printing (bioresin composition and photoexposure parameters). A) Spectral matching between the light source of the DLP printer and the selected photoinitiator; B) absorption spectra of different water-soluble food dyes selected as photoabsorbers to attenuate, but not to block light transmission at 405 ​nm; and C) concentration-dependent spectra of a red food dye (new coccine), selected for this study. D-G) DLP working curves for selected LTS-GelMA and LTS-GelNB bioresins, showing the C_d_ as a function of increasing energy doses and photoabsorber concentration, showing working conditions able to achieve resolution in the z-direction <50 ​μm. H–I) Quantification of the sensitivity of the bioresins to different light outputs (D_p_) and critical energy required to trigger the photo-induced gelation of the polymers, as a function of gelatin and photoabsorber concentrations. In the panels from D to I, data is color-coded to indicate the content of new coccine as photoabsorber following the same legend reported in panel C (pink ​= ​0.03% dye, red ​= ​0.07% dye), with the orange color indicating bioresins without new coccine supplementation. For the data in D) to I), n ​= ​3 for each light dose.

In the design of a resin formulation for DLP printing, the selection of a photoinitiator compatible with the light source of the printer, and of a photoabsorber compound are crucial. In particular, photoabsorbers are intended to limit light penetration beyond the intended crosslinked layer thickness, while not significantly impairing the photocrosslinking reaction [48,49]. The light source of the DLP printer used in this study has a main, narrow emission peak at 405 ​nm, and a secondary peak at 515 ​nm. This overlaps with the broader absorbance peak of the Ru component of the photoinitiators used in this study, which absorbs over the visible light range (400–500 ​nm, with a tail extending beyond 500 ​nm) with a maximum at 450 ​nm (Fig. 3A). Under such blue light stimulation, Ru reacts with SPS to generate free radicals that can be used to trigger both addition polymerization as well as thiol-ene step-growth polymerization [31,37]. In this study, we compared the absorption spectra of three different compounds as candidate biocompatible photoabsorbers, that are commonly used as dyes in the food industry: new coccine, tartrazine and fast green (Fig. 3B). It was observed that 1% fast green, with a primary absorption peak at about 600 ​nm had limited capacity to attenuate light at 405 ​nm, and thus modulate light penetration depth into the bioresin. On the other hand, tartrazine has a prominent absorbance peak at 405 ​nm, which is in the same, desirable absorbance range of Ru, but offers no overlap with the secondary emission peak of the DLP projector, especially if tested at low concentrations needed to not completely block light penetration into the bioresin. This may still induce unwanted radical generation from the photoinitiator system, thus finally affecting printing resolution. Based on the absorbance spectra, we then identified that new coccine is the most suitable photoabsorber as a key component for our bioresin, as it shows a more pronounced absorbance between 450 and 550 ​nm, and a mild attenuation capacity at 405 ​nm, which can be readily modulated in a concentration dependent manner (Fig. 3C). Therefore, we systematically assessed the light curing performance of different new coccine/pre-polymer combinations, and thus drew the working curve for both LTS-GelMA and -GelNB (Fig. 3D – G). While all the hydrogels formulation can technically be printable as long as they can undergo photopolymerization, the expected and achievable printing resolution depends on the response of the material to the supplied light dose. Thus, the working curve determines the range of possible resolutions that can be obtained with a given material. For all the formulations tested, there was a clear trend where increasing new coccine concentration within the bioresin reduced the slope of the working curve. Cure depths (Cd) of ∼50 ​*μ*m could be achieved for both LTS-GelMA and LTS-GelNB by using higher new coccine concentration (0.07% w/v), indicative of higher print resolution. It should also be noted that attempts to print structures with a new coccine concentration higher than 0.07% w/v led to no crosslinking at any of the tested light intensities (data not shown). Consistently, increasing the concentration of new coccine in either 10% or 15% w/v LTS-GelMA led to a decrease in light penetration depth (D_p_) (Fig. 3H), a parameter intrinsic to each resin formulation. Interestingly, this effect was not significantly observed in LTS-GelNB (Fig. 3H), suggesting that the thiol-ene crosslinking mechanism, well-known for its faster kinetics and lower activation threshold compared to acryloyl-based addition polymerization [50], paired with the high reactivity of the Ru-based initiator, made it more difficult to detect subtle changes in the Dp value. In contrast, a higher critical energy (Ec) is required for gel formation when a higher concentration of new coccine was used in both LTS-GelMA or LTS-GelNB bioresins (Fig. 3I), irrespective of the macromer concentration.

### 3.3 Cytocompatibility assessment and DLP printing of LTS-GelMA or LTS-GelNB hydrogel constructs

**Fig. 4:**
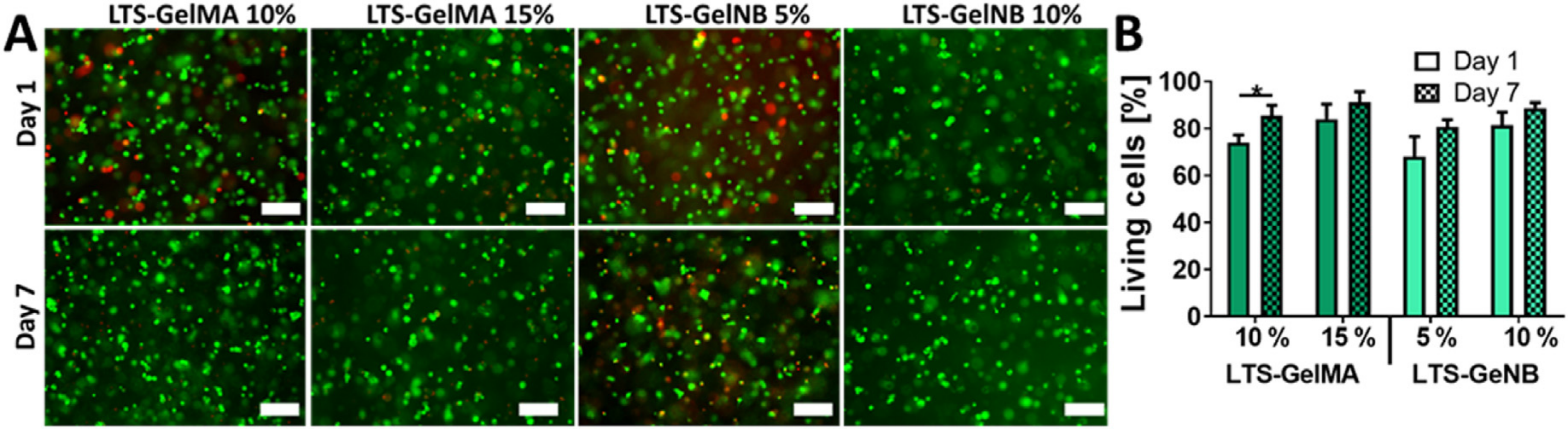
Viability over 7 days of culture for bioprinted bone marrow-derived MSCs. A) LIVE/DEAD staining images obtained *via* fluorescence microscopy, and B) quantitative assessment, showing a ratio of living cells >75% for all formulations and time points, suggesting the overall non-toxicity of the bioresins and printing conditions (∗ ​= ​p ​< ​0.05; n ​= ​3). Scale bars ​= ​200 ​μm.

To ensure the applicability of these bioresins in the fields of bioprinting and tissue engineering, we further encapsulated bone marrow-derived MSCs into LTS-GelMA or LTS-GelNB to perform cytocompatibility assays. For both LTS-GelMA and LTS-GelNB, cells survived the crosslinking process, with excellent cell viability (green cells, Fig. 4A) at the two time points (day 1 and day 7) evaluated. More specifically, at day 1, the samples displayed a percentage of viable cells ranging from 68.02 ​± ​8.60% for 5% w/v GelNB to 84.07 ​± ​6.27% for the 15% w/v GelMA, while at day 7, values ranged from 80.74 ​± ​3.06% (5% w/v GelNB) to 91.10 ​± ​4.51% (15% w/v GelMA) (Fig. 4B). Interestingly, a significant increase in cell viability from day 1 to day 7 was only observed for 10% w/v LTS-GelMA (73.99 ​± ​3.27% at day 1, and 85.46 ​± ​4.45% at day 7, p ​< ​0.05). Thus, no noticeable cytotoxic effect could be detected, in line with previous reports for photo-encapsulation of MSCs in porcine-derived GelMA and GelNB [32], underlining how both LTS-GelMA and LTS-GelNB are cytocompatible bioresins suitable for biofabrication of cell-laden constructs.

As both 10% w/v LTS-GelMA and 5% w/v LTS-GelNB showed comparable compressive moduli between 1 and 2 ​kPa, penetration depth and critical energy, these two bioresin formulations were further selected to perform more complex prints *via* DLP. The comparable mechanical properties were also confirmed in terms of shear modulus, as shown through photorheological characterization. The LTS-GelNB resin exhibited faster gelation kinetics, reaching a storage modulus plateau earlier upon light exposure, when compared to LTS-GelMA. (approximately 4 ​min for GelNB and 7 ​min for GelMA, Supplementary Fig. S2). This faster gelation kinetics profile is comparable with what was previously reported for thiol-norbornene systems [51]. Both hydrogel formulations also showed to be stable upon overnight swelling (Supplementary Fig. S7), and to be susceptible to enzymatic degradation, as tested upon exposure to collagenase (Supplementary Fig. S8), the latter being a necessary requirement for matrix remodelling in tissue engineering applications. However, given the low mechanical properties, it was uncertain whether, even with such soft materials it was possible to resolve minute architectural features with good shape fidelity upon (bio)printing. Hydrogels (including gelatin-derived) displaying compressive moduli as low as ∼1 ​kPa have been previously processed *via* DLP printing, to generate simple structures, such as microgels as injectable carriers for cell delivery [52], an application for which shape retention in convoluted geometries is not necessarily needed. Conversely, structural elements, hydrogel-based microfluidic chips, and perfusable networks, have been produced primarily with hydrogels displaying compressive stiffness in the range of tens to hundreds of kPa [15,21,[53], [54], [55]].

**Fig. 5:**
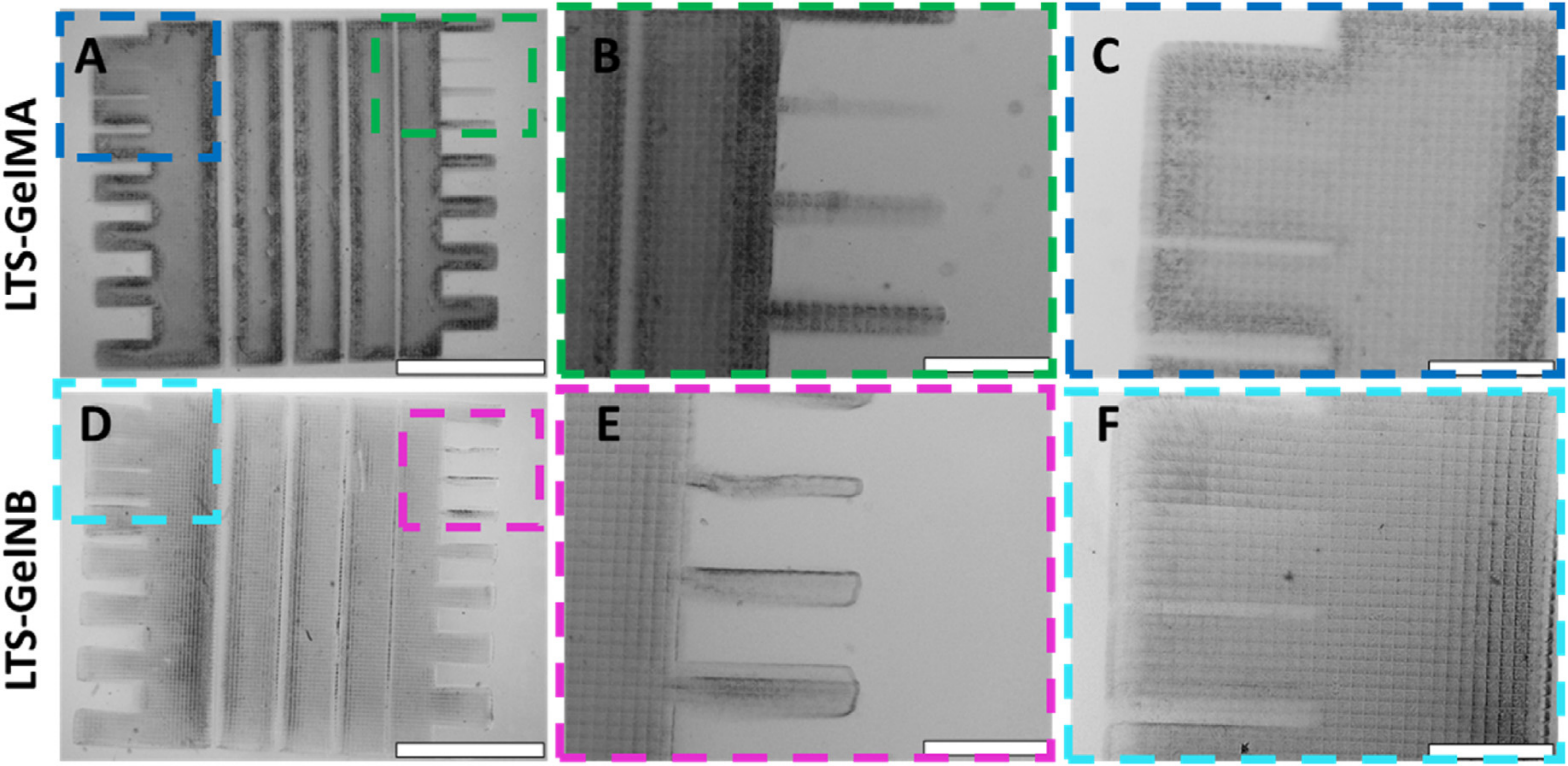
Printed models containing posts and protrusions as well as hollow channels designed with decreasing thickness to assess the ability to resolve positive and negative features in the plane of light projection. Micrographs of A-C) LTS-GelMA and D-F) LTS-GelNB. In panels C and F, it can also be observed how the smallest hollow channels in the design are actually clogged by partially crosslinked material, as can be inferred by the presence of the pixelated pattern within the cavity. Scale bars ​= ​2 ​mm ​(A and D), and ​= ​500 ​μm ​(B,C, E, F).

We firstly evaluated the maximum resolution that could be achieved with the two selected bioresin formulations by printing a simple comb structure. The structure was designed to contain both protrusions and hollow channels with decreasing thickness to evaluate the possibility to form positive and negative features within the hydrogel constructs using DLP printing (Fig. 5). In all printed samples, voxelated surface patterns can be easily recognized, which are a direct result of the light projected from the pixels composing the micromirror device. Solid comb structures of good spatial definition were successfully printed for both LTS-GelMA or LTS-GelNB (Fig. 5A–D). However, a higher resolution was displayed with LTS-GelMA. With this gel, the smallest positive feature obtained was 65 ​± ​14 ​μm as opposed to 76 ​± ​18 ​μm achieved with LTS-GelNB, although this difference was not statistically significant (Table 1, Fig. 5B–E, Supplementary Fig. S9). It should be noted that while the resin and printer described in this study allow printing of pixel-wide positive features, given the low mechanical properties of these bioresins, such thin structures are more likely to be damaged during handling of the prints or during the necessary washing steps. Additionally, LTS-GelMA also resulted in significantly smaller negative features (open channel) of 64 ​± ​7 ​μm in comparison to 95 ​± ​21 ​μm achieved with LTS-GelNB, when aiming to obtain a 100 ​μm channel, indicative of a slightly higher overcrosslinking of the surrounding borders of the cavity, (Table 1, Supplementary Fig. S10), while attempts to resolve channels with theoretical designed sizes between 12.5 and 50 ​μm resulted in partially clogged structures, likely due to the diffusion of reactive species during crosslinking or undesired light scattering (Fig. 5C–F). These results further expand the potential to resolve minute structures when using soft natural origin hydrogels, since to date, single pixel features have been achieved prevalently with stiff, synthetic materials (i.e., PVA-MA [14]). Such stiff resins have also been traditionally needed for resolving open channels with diameters <100–200 ​μm (PEGDA [[56], [57], [58]]). Achieving these intricate features with simple gelatin-based materials, however, has only been possible by developing custom, specialized DLP printers that are engineered to improve resolution by using optics to miniaturize the print itself, thus compromising on the overall size of the achievable objects [59]. In addition, given that direct comparisons between published literature utilizing different DLP and SLA systems for hydrogel printing are difficult due to the lack of homogenous reporting of key printing parameters and print outputs, we are therefore including in the Supplementary Information a table summarizing the main settings and printing results observed in our study and in other relevant, recent references from the literature (Supplementary Table ST1).

**Table 1:** Resolution of printed positive and negative features as obtained by analyzing microscope images. ∗ indicates statistically significant differences between the two bioresins (p ​< ​0.05, n ​= ​3 prints per condition).

Feature	Designed size	LTS-GelMA	LTS-GelNB
Smallest post (positive feature)	50 ​μm	65 ​± ​14 ​μm	76 ​± ​18 ​μm
Smallest open gap∗ (negative feature)	100 ​μm	64 ​± ​7 ​μm	95 ​± ​21 ​μm

**Fig. 6:**
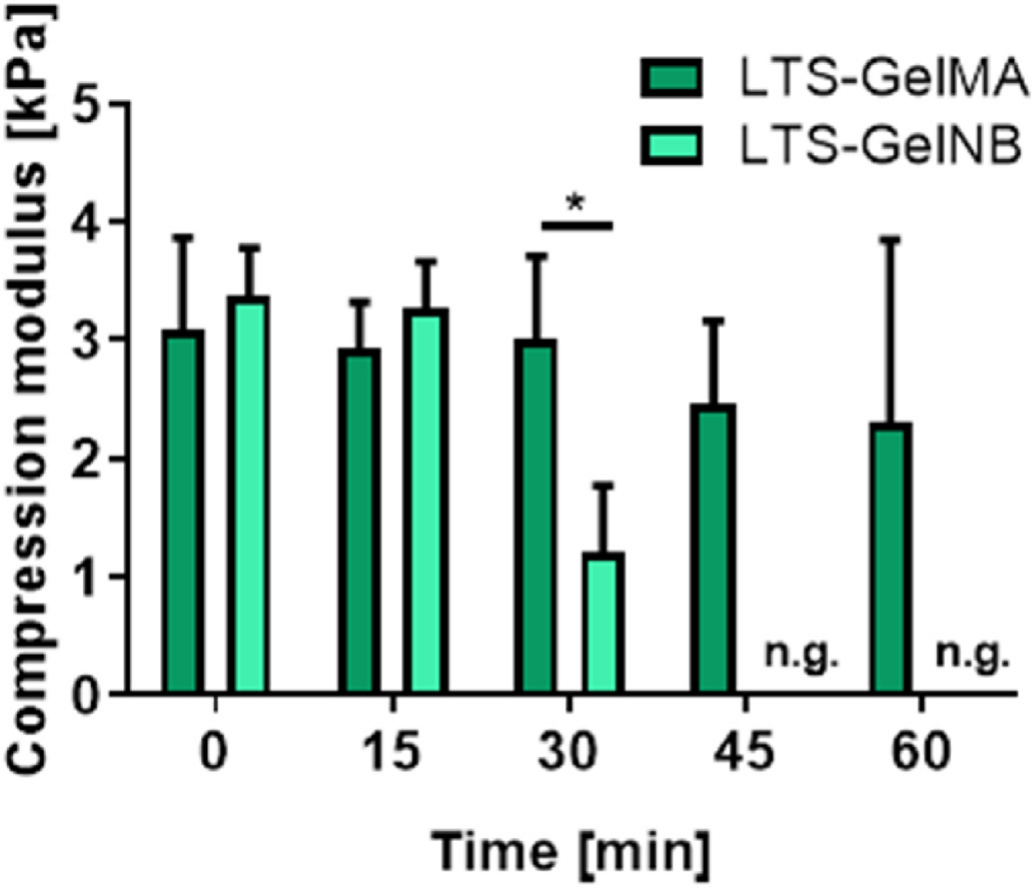
Time window for the initiation and completion of Ru/SPS mediated photocrosslinking of LTS-GelMA (10% w/v) and LTS-GelNB (5% w/v), as estimated measuring the variation of the compressive modulus for gels produced at different latency times after mixing with the photoinitiators. Moduli are normalized against the value found for hydrogels crosslinked right after mixing the photoinitiators. ∗ indicates p ​< ​0.05 (n ​= ​3), n.g. indicates no gelation.

Next, as a preliminary step for 3D printing of larger structures extending in the z-direction, the stability of both LTS-GelMA or LTS-GelNB bioresin was further evaluated. The LTS-GelMA or LTS-GelNB macromer solution were firstly mixed with Ru/SPS and the photoabsorber, then their ability to be printed were tested at every 15 ​min over a time interval up to 1 ​h. It was observed that LTS-GelMA bioresin remained stable over the 1-h window, where all the printed gels yielded similar compressive moduli (Fig. 6). In contrast, the LTS-GelNB bioresin had a limited print window over time, where a significant reduction in compressive modulus was observed after 30 ​min of bioresin preparation, where no gels were formed after 45 ​min of mixing the photoinitiator and photoabsorber into the LTS-GelNB macromer solution. Previous work suggested that Ru/SPS could mediate thiol-persulfate redox reactions even in the absence of light and in a time-dependent fashion [60]. This was likely the reason for the loss of reactivity of the LTS-GelNB resin, due to an early and undesired conversion of norbornene groups. Consequently, despite the proven possibility to print thin structures with resolution in the range of 50–100 ​μm, LTS-GelNB does not appear to have sufficient stability necessary to DLP fabricate structures that require more than 30 ​min to be printed, at least in the formulations reported in this work. With the settings and DLP printer used in this study, 30 ​min translates into approximately 2 ​mm in height. This could be sufficient in several tissue engineering applications, for instance in many organ-on-a-chip applications or when thin regenerative hydrogels need to be fabricated [61].On the other hand, larger 3D structures laden with perfusable cylindrical samples could be produced using LTS-GelMA at 10% w/v (Supplementary Fig. S11), which was thus selected for further investigation in this study.

### 3.4 DLP-bioprinted LTS-GelMA constructs preserve long-term cell functionality

**Fig. 7:**
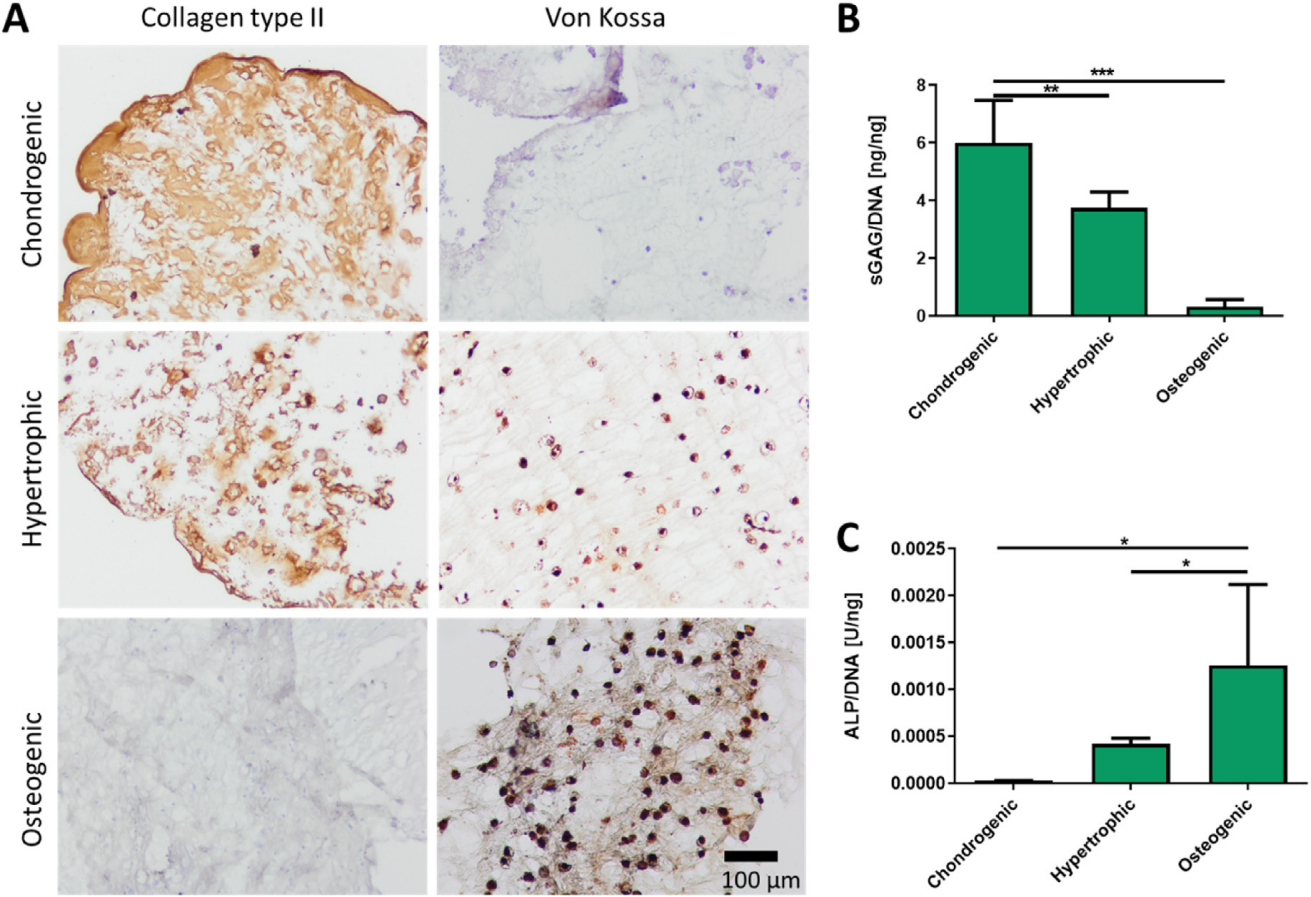
Long-term functionality and preservation of multi-lineage differentiation potential for DLP-bioprinted MSCs in LTS-GelMA, upon 28 days of culture. A) Histological assessment of key cartilage and bone markers (collagen type II and mineral deposits, respectively) for bioprinted MSCs cultured with chondrogenic, hypertrophic and osteogenic differentiation protocols. Quantitative analysis of B) glycosaminoglycan synthesis and C) ALP activity from bioprinted MSCs under the different culture conditions. Scale bar in A ​= ​0.1 ​mm; all photos were taken at the same magnification. ∗ in B) and C) indicate p ​< ​0.05 (n ​= ​3).

Having identified a preferred bioresin formulation from the initial library, we then proceeded to evaluate the long-term survival and functionality of MSCs in DLP-bioprinted LTS-GelMA hydrogel constructs, in particular in the context of bone and cartilage tissue engineering. In that area of research, MSCs have been extensively used for their capacity to directly deposit bone-like and cartilage-like matrix when differentiated into osteoblastic or chondrogenic lineages, respectively [62,63]. In addition, some bone tissue engineering strategies that aim to mimic bone formation paths typical of embryonic development rely on the initial formation of a hypertrophic cartilage template, which can later be remodeled into bone upon implantation *in vivo* [64]. In the bioprinted hydrogel samples, it was observed that the encapsulated MSCs were able to retain their multi-lineage differentiation capability when exposed to media derived from chondrogenic, hypertrophic and osteogenic differentiation protocols. This was evidenced by positive collagen II staining when cultured in chondrogenic or hypertrophic media, and positive von Kossa staining when cultured in hypertrophic or osteogenic media (Fig. 7A). Moreover, ECM deposition appeared diffused throughout the whole hydrogel matrix. Similarly, biochemical analysis provided a quantitative assessment of the capacity of the bioprinted MSCs to secrete tissue-specific biomarkers as a function of the applied differentiation protocol, with high GAG and low ALP synthesis in chondrogenic media (respectively 6.00 ​± ​1.47 ng/ng_DNA_ and ∼2.85 ·10^−5^ U/ng_DNA_), as opposed to those found for the osteogenic media (respectively 0.32 ​± ​0.25 ng/ng_DNA_ and ∼1.26 ·10^−3^ U/ng_DNA_) (Fig. 7B and C). Overall, the hydrogels provided a permissive environment for MSC differentiation and neo-matrix deposition, further suggesting the biocompatibility of the used bioresin components and of the visible-light-induced DLP printing and photocrosslinking steps.

### 3.5 DLP-printing of complex, channel-laden structures using LTS-GelMA bioresins

**Fig. 8:**
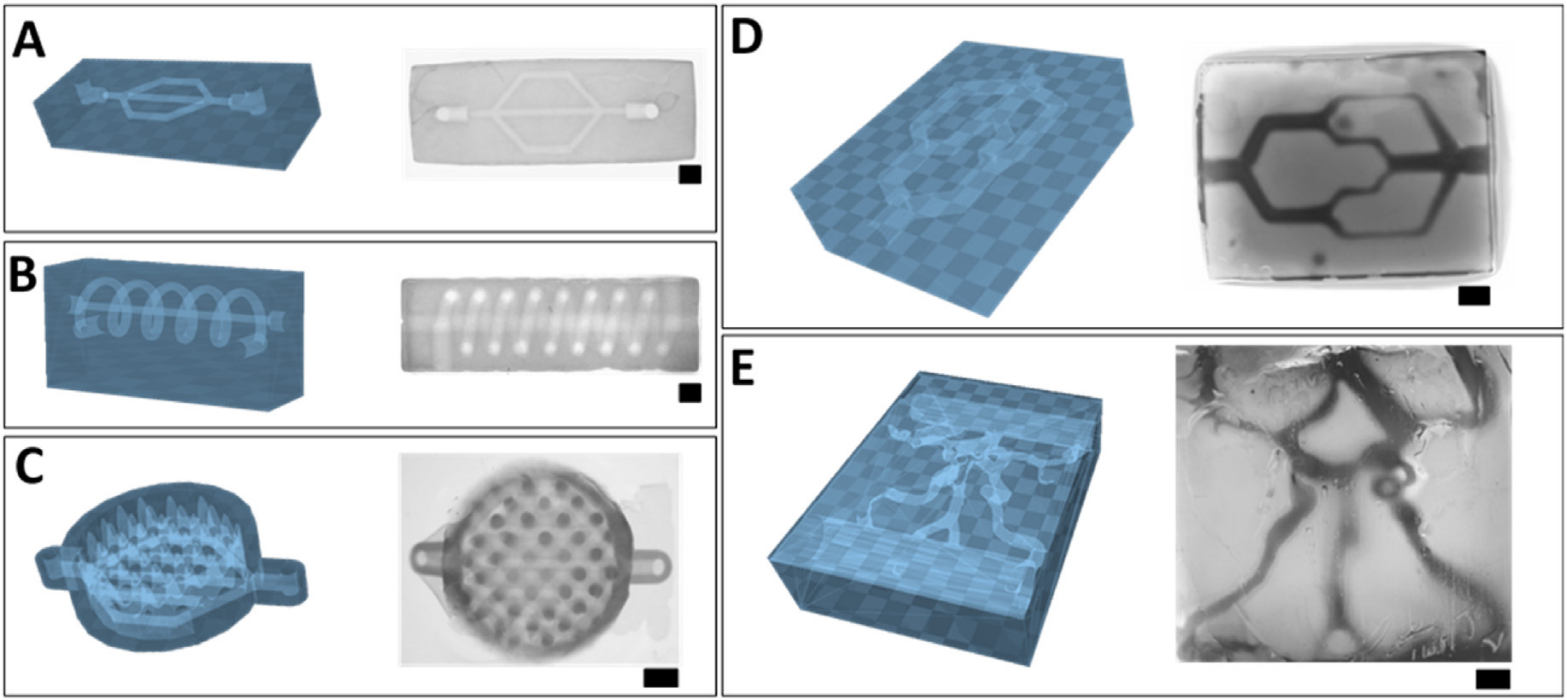
DLP printing of LTS-GelMA hydrogels with embedded complex, perfusable fluidic networks, showing both the STL files (depicted in blue) and the resulting print as imaged *via* stereomicroscopy. A) Branched microfluidic chip, B) spiraling tube wrapped onto a horizontal channel, C) dish with peaks and valleys mimicking the crypt-villi composition of the intestinal epithelium, with a branched network of open channels placed underneath, D) branched microfluidic network with varying channel diameter, and E) anatomical replica based on angiographic 3D data of a portion of the blood vessels within the human Willis circuit, showing the capability to reproduce convoluted, out-of-plane channel networks, with irregular vessel-like channel size. Scale bars ​= ​1 ​mm.

Light-based printing technologies have increasingly drawn attention for their unique ability to reproduce convoluted geometries that are not possible to resolve when using conventional extrusion bioprinting techniques. Notable examples include triply periodic minimal surface (TPMS) structures (including gyroids) [14], and the shaping of vessel and airway-mimetic channel networks within hydrogel blocks, that can also be used to provide nutrients to encapsulated cells [15]. We further examined the feasibility of using the 10% w/v LTS-GelMA bioresin to fabricate complex 3D structures laden with vessel-like channels with increasing architectural complexity (Fig. 8), leveraging its ability to resolve patent channels with a lumen of ∼200 ​μm, even when printed in the direction perpendicular to the direction of light irradiation (Supplementary Fig. S12). Firstly, a hydrogel block with an embedded 3-way microfluidic geometry was successfully printed within a bulk hydrogel replicating the original CAD-design (Fig. 8A). Other complex designs, including a structure with two open channels consisting of a hollow spiral channel wrapped around a horizontal channel were also successfully printed within a bulk LTS-GelMA hydrogel (Fig. 8B), further showcasing the potential of the bioresin. Injection of radiopaque contrast agents provided a visual demonstration of the perfusability of these 3D channels (Supplementary Fig. S13). Given the freedom of design provided by DLP bioprinting, an array of positive features were successfully printed consisting of peaks and valleys mimicking the crypt-villi composition of the intestinal tract, with a branched network of open channels placed underneath (Fig. 8C). High resolution features such as a branched microfluidic network with varying channel diameters (Fig. 8D) and anatomical replica of blood vessels within a region of the human Circle of Willis based on 3D angiographic data (Fig. 8E, Supplementary Fig. S9), were also successfully printed. The possibility to perfuse these structures was proven via the infusion of a colored dye solution, and the smallest tubular feature within the complex Willis vessel network was measured to be 183 ​± ​25 ​μm. Overall, the possibility to generate such high resolution, convoluted structures via vat polymerization printing has not been achieved with other gels with low compressive properties, not even when utilizing other types of low viscosity gelatins obtained from the partial degradation of the polymer backbone [65]. It should be noted that, in the proof-of-concept prints reported in our study, perfusion was demonstrated via manual injection, recent reports in the literature highlight elegant strategies for enabling the connection of hydrogel-based constructs to fluidic tubing and circuits, which could be applied to automate fluid flow [66]. To date complex and branched omnidirectional network of channels have been reported via lithographic printing mainly in stiff synthetic hydrogel, most commonly PEGDA [15] or GelMA and PEGDA blends [28,67], which result in dense networks that limit the range of applications to tissues or models in which cell migration is not needed [68]. Gelatin-only hydrogels, although widely investigated in the field of biofabrication, have been mainly processed to form channels with planar geometries [16,59,[69], [70], [71]]. Out-of-plane branches, highly relevant when addressing the complexity of vascular systems, have been obtained via extrusion printing thanks to the use of sacrificial materials, suspended printing and coaxial nozzles [10,[72], [73], [74]], leveraging on the ability of support materials and viscosity enhancers to preserve the shape of the printed channels. However since prints need to account for the presence of a nozzle, channels forming 3D loops are more difficult to generate with such approaches, and stereolithographic techniques display a superior freedom of design in these cases, as also shown in this present work. Taken together, our results so far showed that unprecedented complex structures can be DLP-printed using the LTS-GelMA bioresin developed in this study. Such anatomical structures could potentially find application in the vascularization of engineered tissues and in organ-on-a-chip devices, for instance in set-ups involving the study of the role of physiological and abnormal blood vessel geometry in vascular diseases, such as thrombotic events [75].

### 3.6 DLP printing of pore-forming LTS-GelMA bioresin

**Fig. 9:**
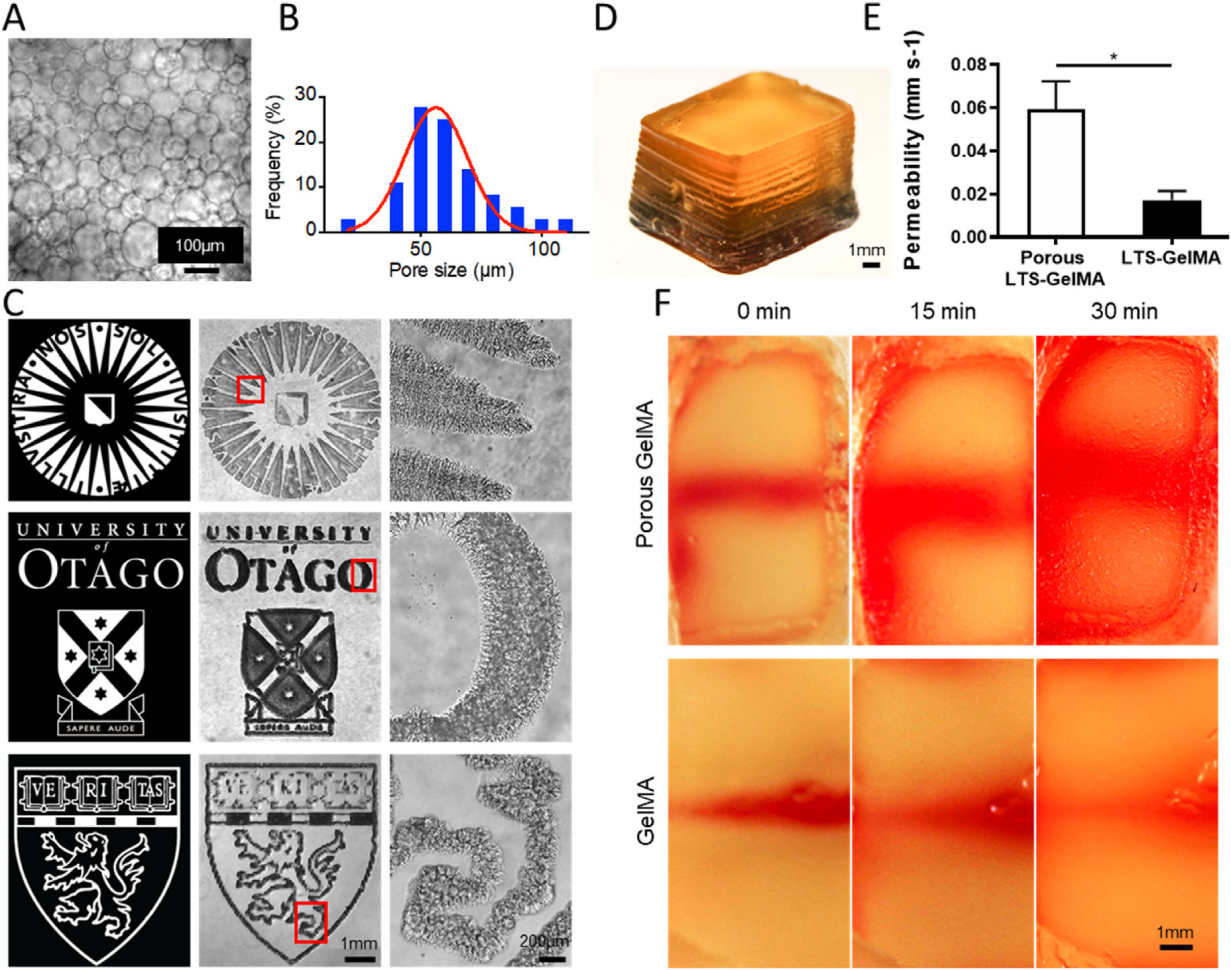
Printing of pore-forming LTS-GelMA bioresin. A) Optical image shows the morphologies of emulsion droplets as the PEO concentration was 1.6% w/v. B) Pore size distribution of PEO droplets in the LTS-GelMA phase. C) Optical micrographs of printed porous LTS-GelMA hydrogel patterns at low magnification and high magnification. D) 3D-printed construct containing a horizontal channel with a 1-mm diameter. E-F) The diffusion of a water-soluble dye in 3D-printed constructs of porous LTS-GelMA hydrogel and standard LTS-GelMA hydrogel. ∗ in E) indicates p ​< ​0.05 (n ​= ​3).

As a final step for the characterization of the LTS-GelMA bioresin, we explored the possibility to modulate the permeability of the hydrogel *via* the introduction of microporosity within the bioprinted structures. Besides working with hydrogels displaying relatively low polymer content and compressive stiffness, in order to enhance cell-material interactions, as well as to promote nutrient transport across a bioprinted construct, a unique ATPE strategy has been recently introduced [[39], [40], [41]]. Here, we utilized PEO as a porogen to emulsify the LTS-GelMA bioresin. The formation of PEO droplets dispersed in the LTS-GelMA phase could be readily observed *via* microscopy, and yielded droplets with an average diameter measuring 71.89 ​± ​17.2 ​μm (Fig. 9A and B), similar to our previous reports where porcine GelMA was used. With this emulsified bioresin, DLP bioprinting was subsequently performed to reproduce the logos of Utrecht University, University of Otago and Harvard Medical School (Fig. 9C). Additionally, to investigate if volumetric structures could also be fabricated, a 3D cubic hydrogel (10-mm length by 6-mm width by 5.6-mm height) was printed featuring a hollow channel of 1 ​mm in diameter in the center (Fig. 9D). When a water-soluble dye was infused in this channel, the pore-forming bioresins demonstrated a dramatic increase (∼3-fold) in the diffusion rate of the dye throughout the whole hydrogel cube, when compared to prints obtained from the standard, unmodified bioresin (Fig. 9E and F). Thus, the possibility to apply this versatile modification to the LTS gelatin bioresin extends the possibilities to modulate the mechanical performance of the resulting hydrogels, and could potentially be further investigated as an approach in the future to promote cell proliferation and colonization of the bioprinted hydrogel structure, as suggested in previous studies involving other biofabrication technologies [39].

## 4 Conclusions

In this study, a novel, visible light responsive gelatin-based bioresin for DLP, which is characterized by its thermal stability in solution at room temperature was developed. These unique thermal properties enable the printing of gelatin on virtually any DLP printer, even in absence of temperature controlling modules. As these are usually not available in most commercially available devices, this offers a generalizable and ready-to-use bioresin formulation. Bioresins based on methacryloyl addition polymerization and thiol-ene photocrosslinking mechanisms both displayed the ability to sustain cell viability upon printing and to resolve and print features in the range of 50 ​μm, even when utilizing prepolymer mixtures yielding hydrogels with low compressive stiffness in the range of 1–2 ​kPa. However, only LTS-GelMA demonstrated sufficient stability over longer printing times, necessary to fabricate larger constructs. Importantly, such optimized LTS-GelMA bioresins permitted the 3D printing of free-form vessel-like and microfluidic-inspired channel networks, enabling to resolve perfusable open channels smaller than 200 ​μm displaying any intricated geometry. In addition, these bioresins are compatible with recently developed strategies to introduce microporosity into the bulk of the hydrogel with the aim to further improve nutrient exchange within a printed gel structure. Combined with the demonstrated ability to sustain over a month of culture the multilineage differentiation ability of bioprinted stem cells, these new bioresins can open new possibilities for hydrogel-based organ-on-a-chip devices, advanced *in vitro* models for disease modelling and drug screening, and for the development of vascularized tissue engineering constructs.

## Credit author statement

Conceptualization: R.L. (Riccardo Levato), K.L. (Khoon Lim), Tim Woodfield (T.W.), Jos Malda (J.M.); Investigation: R.L, K.L, W.L. (Wanlu Li), M.W. (Mian Wang), A.U.A (Ane Urigoitia Asua), L.B.P. (Laura Blanco Peña), Marc Falandt (M.F.), Paulina Nuñez Bernal (P.B.N.); Formal analysis: R.L., K.L., A.U.A. L.B.P., D.G. (Debby Gawlitta), Y.S.Z. (Yu Shrike Zhang). Resources: R.L., J.M.; Visualization: R.L.; Writing – original draft preparation: R.L, K.L.; Writing – review & editing: all co-authors. Project coordination: R.L., K.L., J.M., T.W. Funding acquisition: R.L., J.M. All authors have read and agreed to the published version of the manuscript.

## Declaration of competing interest

The authors declare that they have no known competing financial interests or personal relationships that could have appeared to influence the work reported in this paper.
