# Supplementary material for: High-resolution lithographic biofabrication of hydrogels with complex microchannels from low-temperature-soluble gelatin bioresins
Source: Mater Today Bio. 2021 Nov 19;12:100162. doi: 10.1016/j.mtbio.2021.100162 (PMC8626672; doi:10.1016/j.mtbio.2021.100162)
Supplement: Multimedia component 1 [file mmc1.docx]

**SUPPLEMENTARY INFORMATION**

**High-resolution lithographic biofabrication of hydrogels with complex microchannels from low-temperature-soluble gelatin bioresins**

*Riccardo Levato^1,2,+,*^, Khoon S. Lim^3,+^, Wanlu Li^4^, Ane Urigoitia Asua^2^, Laura Blanco Pe*ña^2^, *Mian Wang^4^, Marc Falandt^1^, Paulina Nu*ñez Bernal^2^, Debby Gawlitta^5^, Yu Shrike Zhang^4^, Tim B.F. Woodfield^3^, Jos Malda^2,1*^

^1^Department of Clinical Sciences, Faculty of Veterinary Medicine, Utrecht University, the Netherlands.

^2^Department of Orthopaedics, University Medical Center Utrecht, The Netherlands.

^3^Christchurch Regenerative Medicine and Tissue Engineering (CReaTE) Group, Department of Orthopaedic Surgery and Musculoskeletal Medicine, University of Otago Christchurch, Christchurch, New Zealand

^4^Division of Engineering in Medicine, Department of Medicine, Brigham and Women’s Hospital, Harvard Medical School, Cambridge, USA

^5^Department of Oral and Maxillofacial Surgery & Special Dental Care, University Medical Center Utrecht, the Netherlands

*^+^*Equal author contribution: R. Levato, K.S. Lim

*Correspondence to: [r.levato@uu.nl](mailto:r.levato@uu.nl) ; [j.malda@umcutrecht.nl](mailto:j.malda@umcutrecht.nl)

**Supplementary Methods**

**Determination of the degree of substitution of the modified gelatins**

For the quantification of the amount of functionalized groups in LTS-GelMA and LTS-GelNB, ^1^H-NMR (400 MHz, Agilent 400 MR-NMR, Agilent Technologies, USA) spectroscopy and a 2,4,6-trinitrobenzenesulfonic acid (TNBSA) assay were performed. Non-modified fish skin gelatin (LTS-Gelatin) was also assessed. For ^1^H-NMR of GelMA, GelNB, and gelatin, the material (40 mg/mL) was dissolved in D_2_O with sodium trimethylsilylpropanesulfonate (DSS) as analytical standard (0.5 mg/mL). The chemical shift of the NMR spectrum was adjusted to the DSS signal (δ , 9H, 0 ppm). From the spectrum, the amount of MA groups in gelMA (AMA_NMR_) and NB groups in GelNB (ANB_NMR_) were calculated with the following equations:

$${AMA}_{NMR} = \frac{\int methacryloyl (peaks at 5.6-5.8 ppm)}{\int DSS (at 0 ppm)} \times\frac{9 (\# protons DSS peak)}{2 (\# of protons MA peak)} \times\frac{0.5 (\frac{mg}{mL}DSS)}{218.32 (Mw of DSS)} \times\frac{1000}{40 (\frac{mg}{mL}of GelMA)}$$

$${ANB}_{NMR} = \frac{\int norbornene endo (peaks at 6.5-6.0 ppm)}{\int DSS (at 0 ppm)} \times\frac{9 (\# protons DSS peak)}{2 (\# of protons NB peak)} \times\frac{0.5 (\frac{mg}{mL}DSS)}{218.32 (Mw of DSS)} \times\frac{1000}{40 (\frac{mg}{mL}of GelNB)}$$

A TNBSA assay was performed for the quantification of the amount of free amine groups present in the gelatin before and after functionalization. A glycine standard curve, to determine the amino group concentration, was prepared with concentrations of 0, 0.8, 8, 16, 32, 64 µg/mL. Gelatin samples were dissolved in 1.6 mg/mL of 0.1 M NaHCO_3_ buffer. Subsequently, 0.5 mL of the sample was mixed with 0.5 mL of a 0.1 (w/v)% TNBSA solution in the buffer and incubated at 37°C for 2 h. Next, the reaction was stopped by the addition of 0.25 mL of 1 M HCl and 0.5 mL of 10 (w/v)% sodium dodecyl sulfate (SDS). The absorbance of the samples was measured by a CLARIOstar Plus® (BMG Labtech, Germany) plate reader at 335 nm. The amount free amines was calculated to be 0.3371 mmol per gram of gelatin, based on the TNBSA results. Finally, the degrees of modification (DM) were calculated via the following equations:

$${DM}_{MA} = \frac{{AMA}_{NMR}}{0.3371} \times100 (\%)$$

$${DM}_{NB} = \frac{{ANB}_{NMR}}{0.3371} \times100 (\%)$$

**Rheological Analysis**

The thermal gelation properties of a 10% w/v solution of cold water fish skin gelatin, LTS-GelMA and LTS-GelNB, were assessed using a DHR2 rheometer (TA Instruments, The Netherlands). Porcine skin gelatin (type A, Sigma-Aldrich, The Netherlands) was also assessed as control. An aluminum flat plate (diameter = 20 mm) with a 200 µm plate gap was used to assess the complex viscosity of the gelatins and hydrogel precursors as a function of time. Measurements were performed at a constant temperature of 21°C, shear rate of 10 rad s-1 and strain of 1.0 % (n = 3). To evaluate the photocrosslinking kinetics of 10% w/v LTS-GelMA and 5% w/v LTS-GelNB, photorheology measurements were carried out using a 20 mm stainless steel flat-plate, and a plate gap of 200 µm. The storage (G’) and loss (G’’) moduli were measured as a function of time as the resin was exposed to a visible light source (1200mha, AOMEES, China). The light was turned on 5 seconds after the start of the measurement. Measurements were performed at a constant temperature of 21°C, shear rate of 10 rad s-1 and strain of 1.0 % (n = 3).

**Enzymatic degradation assay and swelling assay**

In order to assess their susceptibility to enzymatic degradation, photocrosslinked hydrogels (cylindrical samples, diameter = 5 mm , height = 2 mm) obtained from both LTS-GelMA and LTS-GelNB (at the selected concentrations for bioprinting, respectively 10 and 5% w/v), were left to swell overnight in PBS to remove the sol fraction, and subsequently incubated at 37ºC in a 0.2% w/v solution of collagenase type II in Dulbecco’s modified Eagle Medium (DMEM, 31966, Gibco, The Netherlands), supplemented with 10% v/v heat-inactivated fetal bovine serum (FBS Gibco, The Netherlands), and 1% v/v penicillin and streptomycin (Life Technologies, The Netherlands). The cylindrical samples were removed from the enzymatic solution at different time points (10, 20, 30, 45, and 60 min, n =3 per time point), and the mass was measured and compared to that of the hydrogels prior to the incubation in collagenase. In addition, to assess the swelling of the gels after crosslinking, as-casted hydrogel samples (cylindrical samples, diameter = 5 mm , height = 2 mm, n = 3 per gel type) were also incubated in PBS at 37ºC and their wet weight was monitored prior to the incubation and at different time points (8, 16, 24, 32 and 48 hours).

**Supplementary Figures**


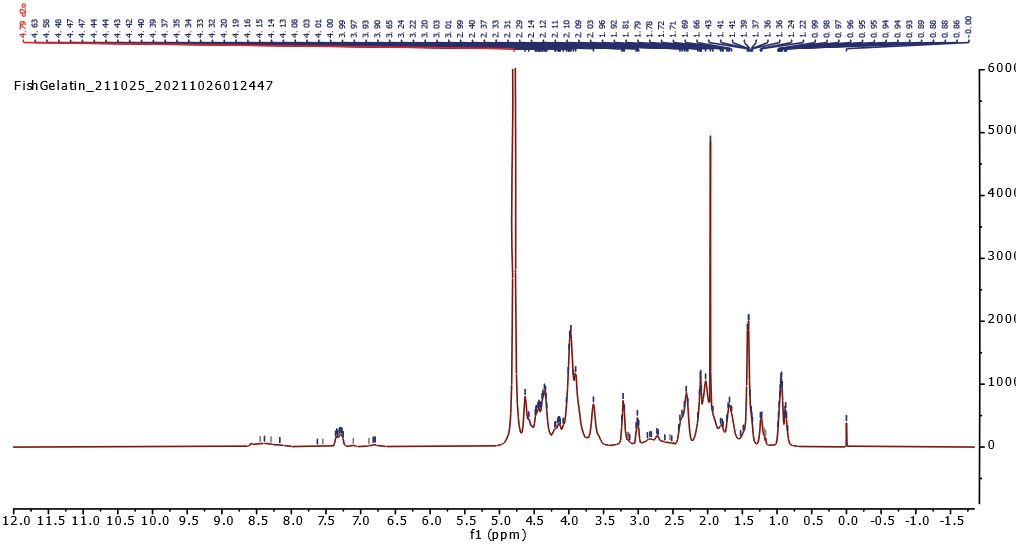


**Figure S1**. ^1^H-NMR spectrum of the unmodified LTS-Gelatin in D_2_O with DSS as analytical standard. ^1^H NMR (400 MHz, d_2_o) δ 8.39 (s, 1H), 7.33 – 7.23 (m, 1H), 4.63 (s, 2H), 4.58 (s, 1H), 4.51 – 4.40 (m, 1H), 4.44 (s, 4H), 4.42 – 4.30 (m, 3H), 4.20 (s, 1H), 4.24 – 4.06 (m, 1H), 4.06 – 3.88 (m, 14H), 3.65 (s, 4H), 3.21 (d, *J* = 7.1 Hz, 2H), 3.01 (t, *J* = 7.5 Hz, 1H), 2.89 – 2.79 (m, 1H), 2.75 – 2.69 (m, 1H), 2.35 (dt, *J* = 33.7, 8.0 Hz, 5H), 2.12 (dd, *J* = 10.9, 5.8 Hz, 2H), 2.03 (s, 7H), 1.96 (s, 1H), 1.92 (s, 5H), 1.84 – 1.76 (m, 1H), 1.75 – 1.63 (m, 4H), 1.51 (d, *J* = 15.6 Hz, 1H), 1.45 – 1.39 (m, 5H), 1.41 – 1.33 (m, 1H), 1.23 (d, *J* = 7.4 Hz, 2H), 0.97 (dd, *J* = 6.8, 3.4 Hz, 1H), 0.98 – 0.86 (m, 3H), 0.86 (t, *J* = 5.7 Hz, 1H).


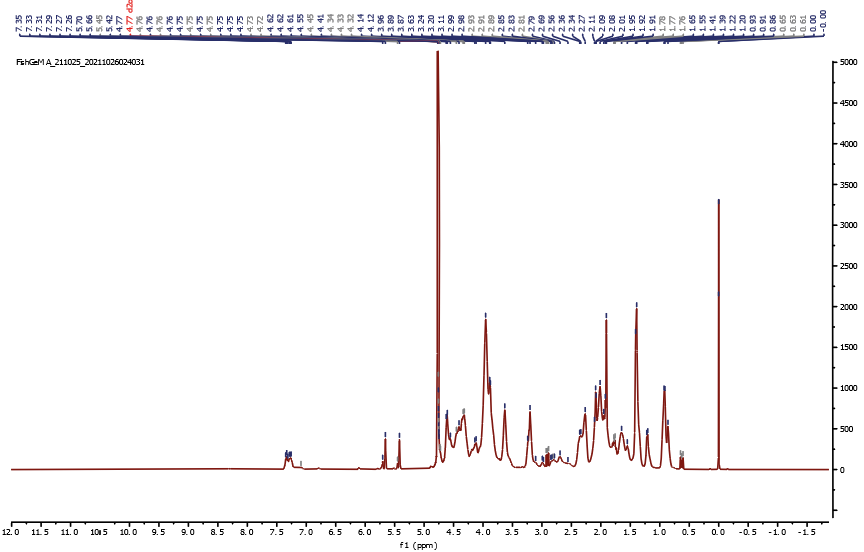


**Figure S2.** ^1^H-NMR spectrum of synthesized LTS-GelMA in D_2_O with DSS as analytical standard. ^1^H NMR (400 MHz, d_2_o) δ 7.37 – 7.23 (m, 2H), 5.66 (s, 1H), 5.42 (s, 1H), 4.77 (s, 20H), 4.61 (s, 5H), 4.13 (d, *J* = 8.2 Hz, 1H), 3.96 (s, 28H), 3.91 – 3.85 (m, 14H), 3.63 (s, 9H), 3.22 (d, *J* = 15.8 Hz, 8H), 2.69 (s, 3H), 2.56 (s, 1H), 2.35 (d, *J* = 7.5 Hz, 2H), 2.27 (s, 6H), 2.08 (d, *J* = 3.3 Hz, 2H), 1.94 (d, *J* = 10.3 Hz, 1H), 1.91 (s, 4H), 1.65 (s, 5H), 1.40 (d, *J* = 7.2 Hz, 18H), 1.21 (d, *J* = 7.0 Hz, 5H), 0.93 (s, 9H), 0.86 (s, 4H).


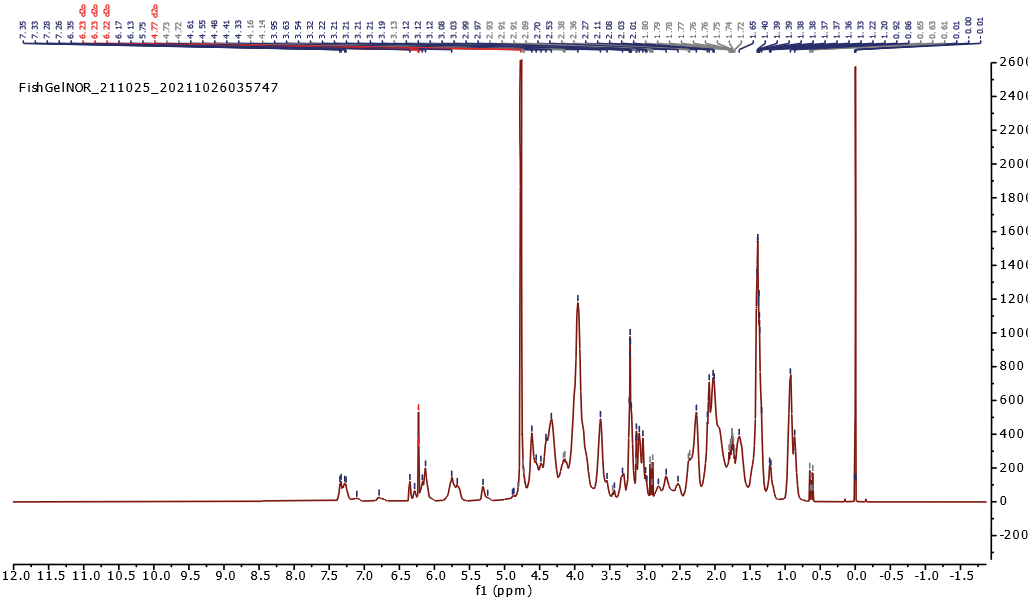


**Figure S3.** ^1^H-NMR spectrum of synthesized fish GelNOR in D_2_O with DSS as analytical standard. ^1^H NMR (400 MHz, d_2_o) δ 7.36 – 7.18 (m, 1H), 6.38 – 6.06 (m, 1H), 5.71 (d, *J* = 31.4 Hz, 1H), 5.31 (s, 0H), 4.77 (s, 29H), 4.61 (s, 1H), 4.33 (s, 4H), 3.95 (s, 10H), 3.63 (s, 3H), 3.24 – 3.17 (m, 2H), 3.08 (s, 1H), 3.01 (d, *J* = 15.2 Hz, 1H), 2.68 (t, *J* = 56.0 Hz, 1H), 2.27 (s, 2H), 2.16 – 1.84 (m, 1H), 1.65 (s, 2H), 1.42 – 1.31 (m, 6H), 1.24 – 1.18 (m, 1H), 0.92 (s, 2H), 0.86 (s, 1H).

**Figure S4.** Complex viscosity as a function of time of 10% w/v solutions of porcine gelatin, LTS-Gelatin, LTS-GelMA and LTS-GelNB at room temperature (21°C) demonstrate the absence of thermally-induced gelation of LTS samples at the printing temperature.

**Figure S5**. Representative stress-strain curve of different hydrogels based on GelMA and GelNB, showing the increasing stiffness as a function of polymer concentration, as well as the broader range of mechanical properties that are covered by the GelNB formulations.


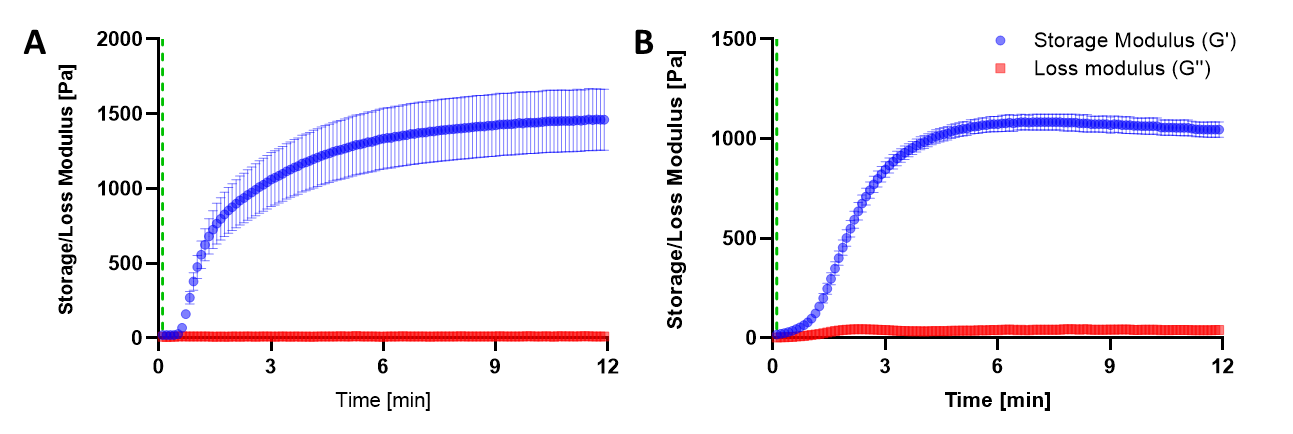


**Figure S6**. Crosslinking kinetics of A) LTS-GelMA 10% w/v and B) LTS-GelNB 5%, showing the faster completion of the reaction and reaching of the storage modulus plateau by the thiol-ene crosslinked bioresin (n=3).

**Figure S7**. Exposure to a collagenase solution demonstrating the gradual degradation of the hydrogels over time. Data is represented as mass percentage with respect to the gels prior to the incubation in the collagenase solution. Both hydrogel types were completely degraded at the 60 minutes mark in this accelerated degradation assay (* = p < 0.05, n=3).

**Figure S8.** Evolution of the wet weight of the hydrogels over time upon incubation in PBS, normalized against the wet weight of casted gels at time point 0 (swelling ratio). The swelling ratio remained constant across different time points from 8 hours onwards (no statistically significant difference, n=3).

**Figure S9.** Optical micrograph of a magnification of the smallest printable positive feature in LTS-GelMA bioresins, showing single pixel posts. Scale bar = 250 µm.


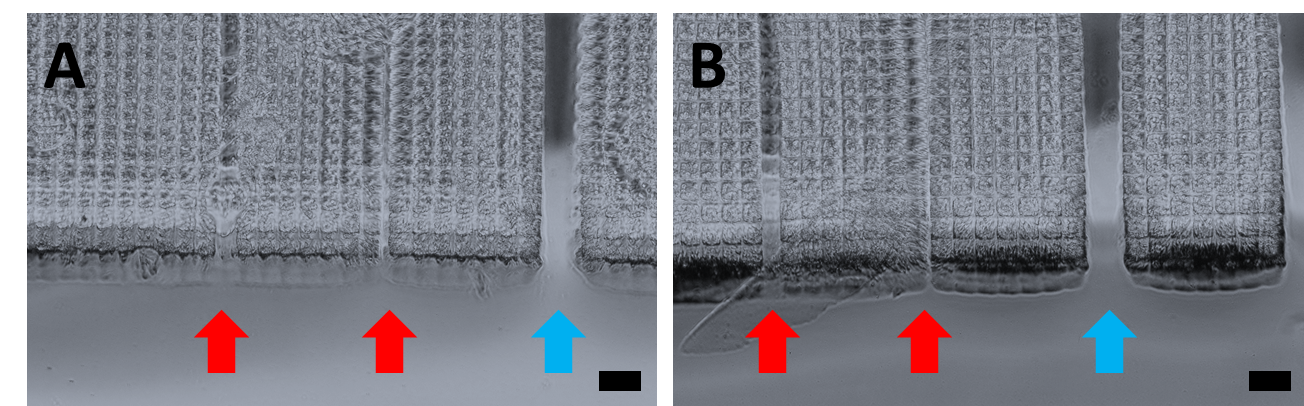


**Figure S10**: Representative micrograph illustrating the smallest fully open channel that could be resolved with the (A) LTS-GelMA 10% w/v and with the (B) LTS-GelNB 5% w/v bioresins. Red arrows point towards channels clogged by partly crosslinked resin, while blue arrows point towards fully open channels. Scale bars = 100 µm.

**
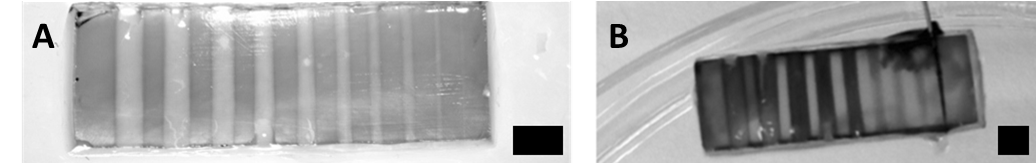
**

**Figure S11.** (A, B) Perfusable, open channels with diameter down to 200 µm as printed in LTS-GelMA in the direction parallel to the plane of light projection. Scale bars = 2 mm.


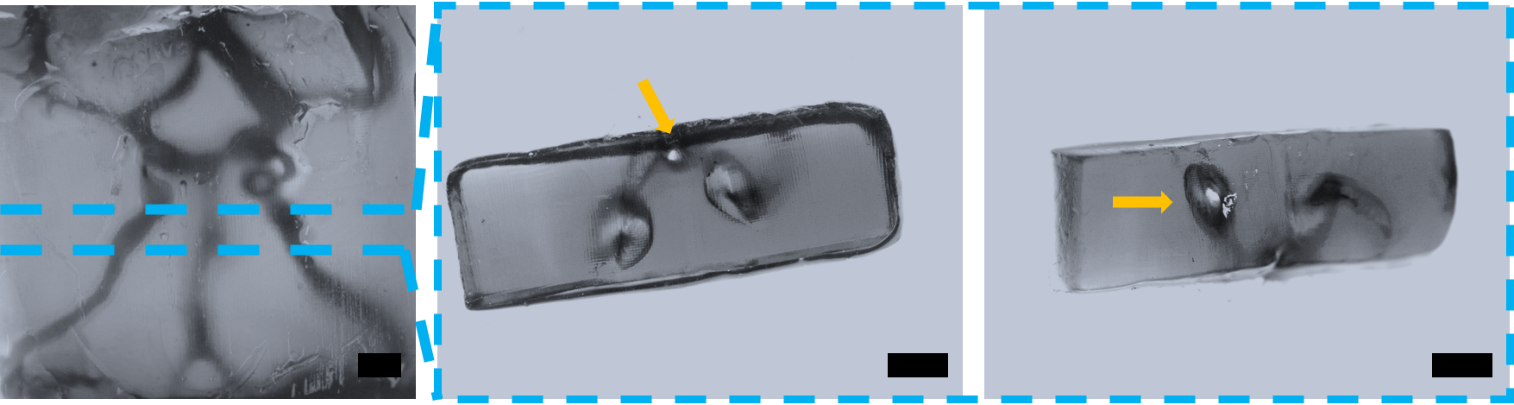


**Figure S12.** Cross sectional views of a hydrogel construct embedding a complex vessel network mimicking the Willis circuit, as printed from LTS-GelMA. Samples were first infused with an Alcian Blue dyed solution, which was subsequently washed out by further injection of PBS, leaving a slight staining at the wall of the channels that facilitates visualization and contrast upon imaging. Given the convoluted geometry of the channels and the irregular diameter along the length of the vessel that replicates the native anatomy, the open lumen (highlighted by the orange arrows) can be readily appreciated only when the sections are visualized at specific angles. Scale bars = 1 mm.

**Figure S13.** Spiraling channel-straight vessel couple within an LTS-GelMA printed gel. A) Rendering from the designed STL file, B) stereomicroscopic image, and C) false color virtual reconstruction from µCT scans, upon infusion of radiopaque contrast agents within both channels, marking the perfusability of the printed convoluted fluidic vessels. Scale bar in B = 2 mm.

**Supplementary Tables**

**Supplementary Table ST1**. Summary of printer settings and main results from our work and from recent papers involving lithographic biofabrication. The numbers in the Reference column refer correspond to the same order as reported in the main manuscript to which this Supplementary file belongs.

| **Material and photoinitiator** | **Printer type, light source, intensity and exposure per layer** | **Out-of-plane**  **channel networks** | **Main results** | **Reference** |
| --- | --- | --- | --- | --- |
| GelMA and GelNB, Ru/SPS | EnvisionTec Perfactory 3 mini, 405 nm light, 6.5 mW/cm^2^, 10 seconds | Yes | Bioresin with compressive modulus between 1 and 2 kPa. Positive and negative features ⁓65 µm. Perfusable 3D convoluted channel networks, diameter ⁓183 µm. Long term culture (28 days), bone and cartilage differentiation post-printing | This study |
| GelMA, PEGDA, LAP | Custom-made, paired with microfluidic system, 365 nm, 100 mW/cm^2^, 0.1-5seconds | No, in-plane complex vessel networks only | Thickness set to 100µm, xy resolution ⁓10µm. Mechanical properties not reported. Multimaterial prints can be readily incorporated thanks to the microfluidic set-up. | [11] |
| PVA-MA, Ru/SPS | EnvisionTec Perfactory 3 mini, 405 nm light, 7.25 mW/cm^2^, 10 seconds | No, but porous gyroids were printed | Bioresin with compressive modulus of ⁓50 kPa. Positive features ⁓50 µm. Addition of 1% GelMA needed to ensure cell viability >90% | [14] |
| PEGDA and GelMA, LAP | Lumen X (Volumetric Inc), 405 nm, not reported | Yes | Compression modulus not measured, but estimated storage modulus from rheometry ⁓20-35kPa. Perfusable channel networks diameter ⁓400µm. Gas exchange demonstrated. | [15] |
| 4-arms PEGDA, with added GelMA, LAP | Miicraft DLP printer, 365 nm, 6 mW/cm^2^, 5 to 20 seconds. | Yes, vessels (diameter>1mm) in a large heart-shaped model | Storage modulus ⁓13kPa  Smallest channel diameter ⁓500µm. high cell viability 14 days post printing (>90%). | [55] |
| PEGDA, TPO | Custom-made, exposure conditions not reported | No, only 2D-like vessels | Bioresin mechanical properties or PEGDA concentration not reported. Smallest channel in the xy plane ⁓32µm. No cell bioprinting. | [56] |
| PEGDA, LAP | Custom-made, 365 nm, 10.9 mW/cm^2^, 15 seconds | Yes, channels with square profile | Shear modulus ⁓400 kPa. Channels with ⁓100x100µm size profile. | [57] |
| PEGDA, Irgacure-819 | Custom-made, 385 nm and 405nm, exposure settings not reported | No | Resolution not measured, but 1x1mm channels were printed, with an upper wall as thin as ⁓50µm | [58] |
| GelMA, GM-hyaluronan, LAP | Custom-made, UV-light, 88 mW/cm^2^ | No, semi-2D hexagonal channel projections | Compressive modulus ⁓4 kPa. Smallest cavity ⁓40µm. Construct height limited to 200µm. Liver cytochromes upregulated when tri-cultures of iPSC-derived hepatocytes, HUVECs and adipose-derived stem cells. | [59] |
